# Epigenetic dysregulation-mediated COL12A1 upregulation predicts worse outcome in intrahepatic cholangiocarcinoma patients

**DOI:** 10.1186/s13148-022-01413-5

**Published:** 2023-01-24

**Authors:** Zengwei Tang, Yuan Yang, Qi Zhang, Tingbo Liang

**Affiliations:** 1 Department of Hepatobiliary and Pancreatic Surgery, The First Affiliated Hospital, School of Medicine, Zhejiang University, Hangzhou, 310003 Zhejiang China; 2 Zhejiang Provincial Key Laboratory of Pancreatic Disease, The First Affiliated Hospital, School of Medicine, Zhejiang University, Hangzhou, 310003 Zhejiang China; 3 Zhejiang Clinical Research Center of Hepatobiliary and Pancreatic Diseases, Hangzhou, 310003 Zhejiang China; 4 Department of Hematology, Peking Union Medical College Hospital, Chinese Academy of Medical Sciences and Peking Union Medical College, Beijing, 100730 China; 5 Zhejiang University Cancer Center, Hangzhou, 310058 Zhejiang China

**Keywords:** Intrahepatic cholangiocarcinoma, COL12A1, Epigenetics, miR-424-5p, Aberrant hypermethylation

## Abstract

**Background:**

Collagen type XII alpha 1 chain (COL12A1) is associated with human cancer progression. Nevertheless, the expression pattern and the function of COL12A1 in intrahepatic cholangiocarcinoma (iCCA) remain unknown. The present study was performed to assess the role of COL12A1 in iCCA.

**Results:**

A total of 1669 genes, differentially expressed between iCCA and nontumor liver tissue samples, were identified as potential tumor-specific biomarkers for iCCA patients. Of these, COL12A1 was significantly upregulated in clinical iCCA tissue samples and correlated with epithelial–mesenchymal transition gene set enrichment score and advanced tumor stage in clinical iCCA. COL12A1-high expression was associated with the poor prognoses of iCCA patients (*n* = 421) from four independent cohorts. Promoter hypermethylation-induced downregulation of miR-424-5p resulted in COL12A1 upregulation in clinical iCCA. Experimental knockout of COL12A1 inhibited the proliferation, invasiveness and growth of iCCA cells. MiR-424-5p had a therapeutic potential in iCCA via directly targeting COL12A1.

**Conclusions:**

Promoter hypermethylation-induced miR-424-5p downregulation contributes to COL12A1 upregulation in iCCA. COL12A1 is a promising druggable target for epigenetic therapy of iCCA.

**Graphical Abstract:**

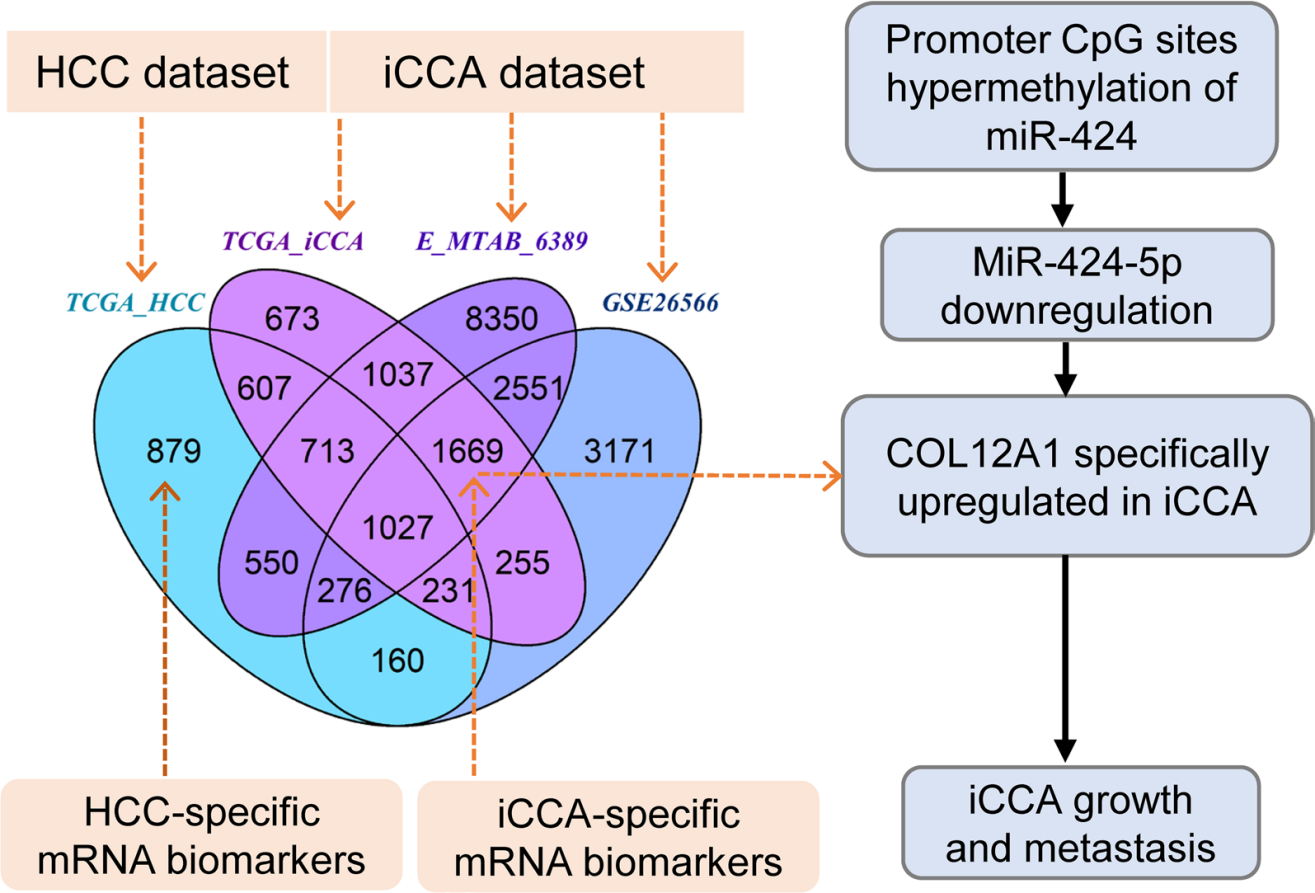

**Supplementary Information:**

The online version contains supplementary material available at 10.1186/s13148-022-01413-5.

## Background

Intrahepatic cholangiocarcinoma (iCCA) is a highly aggressive malignancy with dismal prognosis [1–3]. Its incidence and mortality are growing worldwide in recent years [4]. Due to lack of disease-specific biomarker for early detection of iCCA, most iCCA patients are typically diagnosed at an advanced stage when the currently available systemic therapies have limited effectiveness [5]. Therefore, identification of tumor-specific biomarkers with druggable targets is urgently needed to discover new therapeutic agents for personalized therapy of iCCA.

Genome and transcriptome sequencing had provided valuable insights into the molecular etiologies of iCCA in the past decade [6–9]. Accordingly, several molecular inhibitors binding to fibroblast growth factor receptor (FGFR) 2 or isocitrate dehydrogenase 1 or 2 (IDH1 or 2) were developed for management of advanced iCCA carrying FGFR2 fusion or IDH1/2 mutation [10]. Recent research provided new insights into molecular pathogenesis of iCCA at proteomic scale [11]. Nevertheless, there is still lack of research works to reveal common hallmarks and tumor-specific molecular events in iCCA from different omics datasets.

To reveal common molecular signatures involved in iCCA development, we performed an integrative analysis of multiple omics datasets including liver cancer samples from different centers. Furthermore, among the identified potential tumor-specific biomarkers, we investigated the expression pattern, clinical relevance, biological function and upstream regulatory mechanism of collagen type XII alpha 1 chain (COL12A1) in iCCA.

## Results

### Identification of potential tumor-specific mRNA biomarkers for iCCA patients

**Fig. 1:**
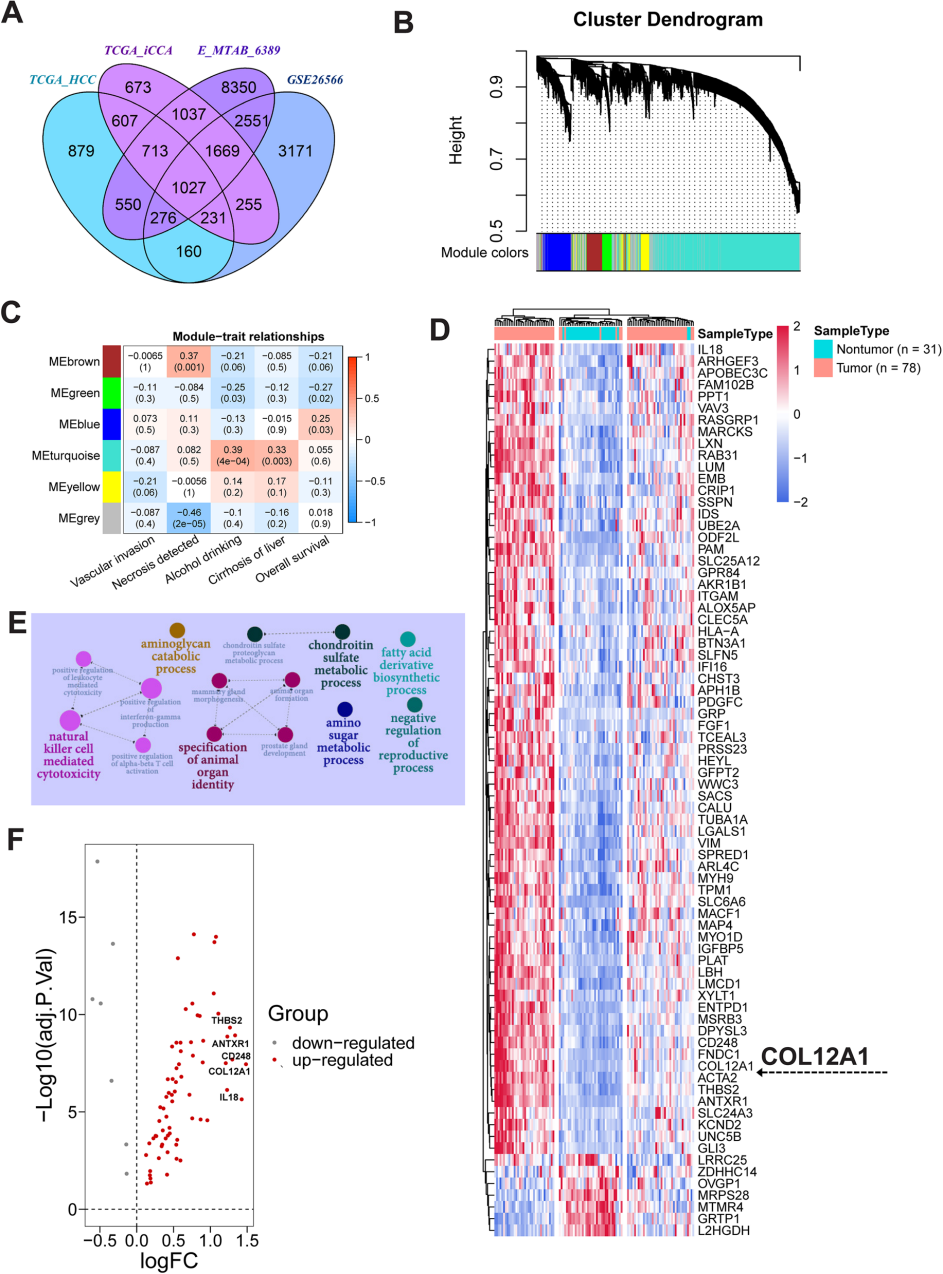
Identification of potential mRNA biomarkers for iCCA patients. **A** Venn plot showing iCCA/HCC-specific mRNA biomarkers among differentially expressed genes between nontumor liver tissue and iCCA or HCC tissue samples from TCGA, GEO or ArrayExpress datasets, respectively. TCGA-HCC dataset including 50 nontumor liver tissue samples and 371 HCC tissue samples, TCGA-iCCA dataset including 8 nontumor liver tissue samples and 32 iCCA tissue samples, E-MTAB-6389 dataset including 31 nontumor liver tissue samples and 78 iCCA tissue samples, and GSE26566 dataset including 65 nontumor liver tissue samples and 104 iCCA tissue samples. Gallbladder or extrahepatic carcinoma samples (*n* = 4) in TCGA cholangiocarcinoma dataset were excluded from our analysis. **B** Hierarchical clustering dendrogram showing gene expression modules in E-MTAB-6389 dataset including 1669 iCCA-specific mRNA biomarkers. **C** Heatmap to show the correlation between gene expression modules and clinical features of patients with iCCA enrolled in E-MTAB-6389 cohort. Value represents coefficient of Pearson correlation test, and the number in parentheses denotes statistical significance. **D** Heatmap to show the expression patterns of iCCA-specific mRNA biomarkers from brown module (MEbrown) in iCCA and nontumor liver tissue samples from E-MTAB-6389 dataset. **E** GO enrichment analysis showing biological processes significantly enriched by iCCA-specific mRNA biomarkers from brown module. **F** Volcano plot to show the expression fold change of 76 differentially expressed genes from brown module. *iCCA*, intrahepatic cholangiocarcinoma; *HCC*, hepatocellular carcinoma; *GEO*, Gene Expression Omnibus; *TCGA*, The Cancer Genome Atlas

Differential analysis showed that 2696 human genes were aberrantly expressed across three independent iCCA datasets (Fig. 1A). Of these, 1027 genes were also aberrantly expressed in hepatocellular carcinoma (HCC) samples from TCGA dataset, and the remaining 1669 genes were identified as potential iCCA-specific mRNA biomarkers (Fig. 1A). Weighted correlation network analysis (WGCNA) showed that the expression profiles of these potential tumor-specific biomarkers clustered into 6 gene expression modules (Fig. 1B). Among these, we are interested in the brown module (MEbrown) as it was positively correlated with necrosis detected in iCCA tumor and inversely correlated overall survival time of iCCA patients from E-MTAB-6389 cohort (Fig. 1C). Cluster analysis of a total of 76 genes from brown module showed that, except for 7 mRNA biomarkers (LRRC25, ZDHHC14, OVGP1, MRPS28, MTMR4, GRTP1 and L2HGDH) downregulated in iCCA, the remaining detected mRNA biomarkers were markedly upregulated in iCCA tissue samples relative to nontumor liver tissue samples from E-MTAB-6389 cohort (Fig. 1D).

Enrichment analysis showed that the identified mRNA biomarkers are significantly enriched in 7 main biological processes including natural killer cell mediated cytotoxicity, aminoglycan catabolic process, fatty acid derivative biosynthetic process, specification of animal organ identity, chondroitin sulfate metabolic process, amino sugar metabolic process and negative regulation of reproductive process (Fig. 1E). Volcano plot showing that COL12A1, IL8, CD248, ANTXR1 and THBS2 were the top five upregulated genes from brown module (Fig. 1F). Given iCCA is a collagen-rich malignancy, and the expression fold change of COL12A1 ranked first among the identified differentially expressed genes from brown module (Fig. 1F). We subsequently investigated and validated the expression profile, clinical significance, upstream regulatory mechanism and the biological role of COL12A1 in iCCA.

### The upregulation of COL12A1 in clinical iCCA tissues

**Fig. 2:**
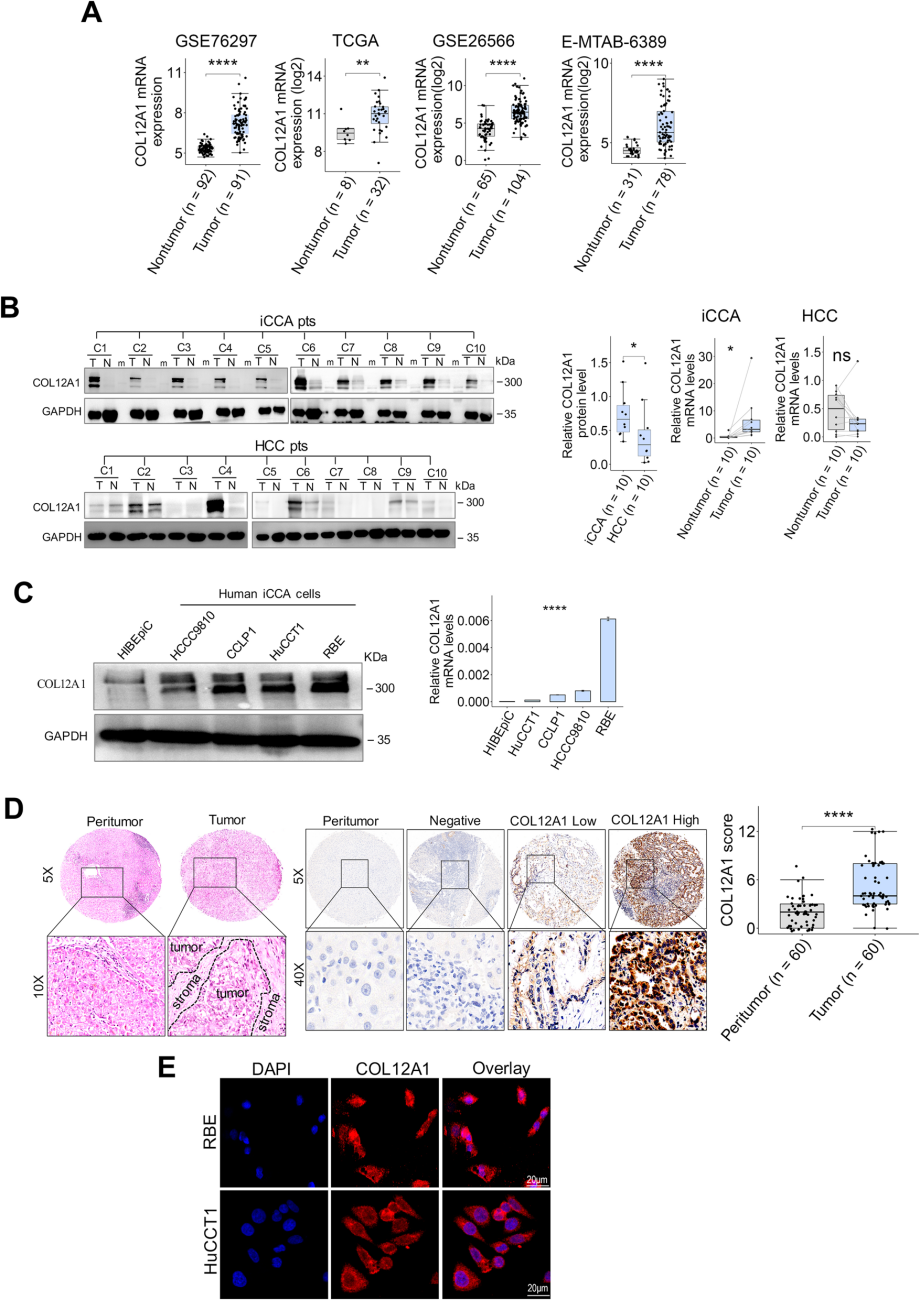
COL12A1 is upregulated in clinical iCCA tissues and human iCCA cells. **A** Box plots showing COL12A1 mRNA levels in iCCA and adjacent nontumor liver tissues from TCGA, GEO or ArrayExpress datasets, respectively. **p < 0.01; ****p < 0.0001, using two-tailed unpaired Student's *t* test. **B** COL12A1 expression levels in iCCA, HCC and the paired nontumor liver tissues were determined by immunoblotting and RT-PCR, respectively. ns, *p* ≥ 0.05. *p < 0.05, using two-tailed paired Student's *t* test except for the comparison of the relative COL12A1 protein level between iCCA and HCC tissues using Wilcoxon test. **C** COL12A1 expression pattern in human iCCA and normal human intrahepatic biliary cell (HIBEpiC) lines were determined by immunoblotting and RT-PCR, respectively. ****p < 0.0001, using one-way analysis of variance (ANOVA). **D** Representative images of H&E and COL12A1 staining in 60 pairs of iCCA samples. Boxplot showing COL12A1 protein levels in iCCA and the paired nontumor liver tissues. ****p < 0.0001, using two-tailed paired Student's *t* test. **E** Representative images of immunofluorescence staining for COL12A1 and DAPI in the indicated human iCCA cells. Original magnification: 100X; scale bar = 20 μm. Experiments were triplicated. *iCCA*, intrahepatic cholangiocarcinoma;* HCC*, hepatocellular carcinoma; *GEO*, Gene Expression Omnibus; *TCGA*, The Cancer Genome Atlas; *RT-PCR*, real-time PCR; *T*, tumor tissue; *N*, paired nontumor liver tissue, *m*: protein maker (ladder)

Reanalysis of multiple transcriptomic datasets revealed that COL12A1 mRNA was significantly upregulated in clinical iCCA samples relative to the adjacent nontumor liver tissue or HCC tissue samples (Fig. 2A, Additional file 1: Fig. S1A). Consistent with the findings in omics datasets, immunoblotting and RT-PCR confirmed that COL12A1 mRNA and protein were significantly overexpressed in clinical iCCA tissue specimens (Fig. 2B). In addition, COL12A1 mRNA and protein were upregulated in the indicated human iCCA cells in comparison with normal intrahepatic bile duct cells (Fig. 2C). Furthermore, COL12A1 mRNA and protein expression was similar between clinical HCC tissue and nontumor liver tissue samples (Fig. 2B, Additional file 1: Fig. S1B), whereas COL12A1 protein was downregulated in human HCC cell lines relative to normal liver cell line HL7702 (Fig. S1C). IHC and immunofluorescence staining showed that COL12A1 protein was expressed in both cytoplasm and nucleus of iCCA cells, as well as the extracellular matrix of clinical iCCA tissue (Fig. 2D, E).

### COL12A1 upregulation associated with poor clinical outcomes in iCCA patients

**Fig. 3:**
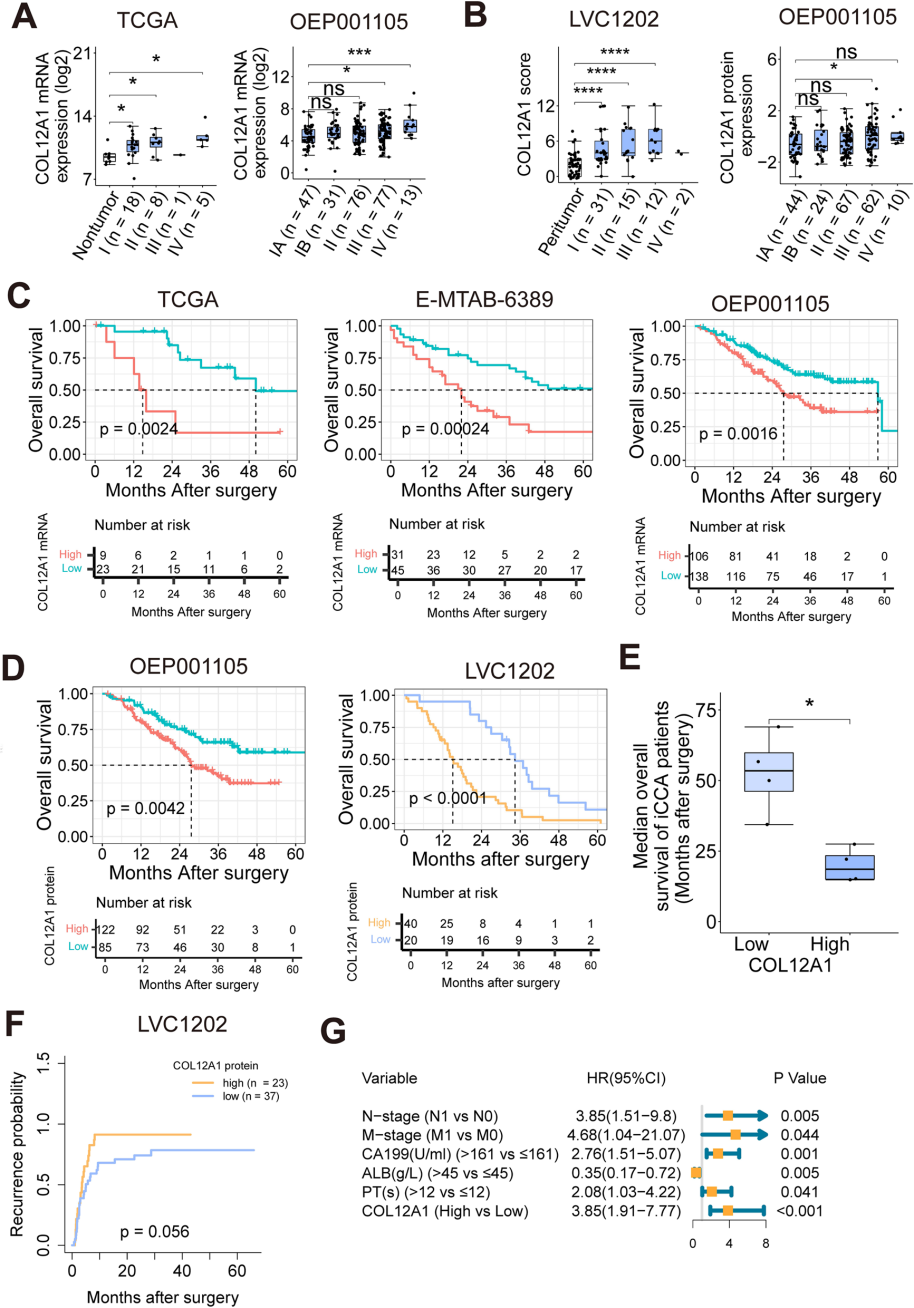
COL12A1 upregulation predicts worse clinical outcomes in iCCA patients. **A** Boxplots to show COL12A1 mRNA levels in adjacent nontumor and tumor specimens stratified by TNM stage (Stage I to Stage IV) from TCGA or OEP001105 iCCA cohort. ns, *p* ≥ 0.05. *p < 0.05, ***p < 0.001, based on Bonferroni post-tests. **B** Boxplots to show COL12A1 protein levels in adjacent nontumor and tumor specimens stratified by TNM stage (Stage I to Stage IV) from LVC1202 or OEP001105 cohort. ns, *p* ≥ 0.05. *p < 0.05, ****p < 0.001, based on Bonferroni post-tests. **C** Overall survival curves stratified by COL12A1 mRNA expression levels in iCCA patients included in TCGA, E-MTAB-6389 or OEP001105 cohort, respectively. *p* is based on log-rank test. **D** Overall survival curves stratified by COL12A1 protein expression levels in two independent iCCA cohorts (OEP001105 and LVC1202). *p* is based on log-rank test. **E** Boxplot to show the median overall survival time of iCCA patients with COL12A1-high/low expression from TCGA, E-MTAB-6389, OEP001105 and LVC1202 cohorts. *p < 0.05, based on two-tailed unpaired Student's *t* test. **F** Recurrence probability curves stratified by the expression level of COL12A1 in iCCA patients. *p* is based on log-rank test. **G** Forest plot showing multivariate survival analysis result from the Cox’s regression model. TCGA-iCCA cohort is from The Cancer Genome Atlas (TCGA); E-MTAB-6389 iCCA cohort is from ArrayExpress; OEP001105 iCCA cohort is from National Omics Data Encyclopedia (NODE). *iCCA* intrahepatic cholangiocarcinoma, *TCGA* The Cancer Genome Atlas

COL12A1 expression was markedly increased in iCCA patients at the advanced stage from TCGA, OEP001105 or LVC1202 cohorts (Additional file 7: Table S1, Fig. 3A, B). Survival analyses showed that iCCA patients with high COL12A1 mRNA expression had a significant worse prognosis than iCCA patients with low COL12A1 mRNA expression from TCGA, E-MTAB-6389 or OEP001105 cohort (Fig. 3C), and the corresponding optimal cutoff value for dividing iCCA patients into COL12A1 mRNA-high/low expression (log2 expression) group was 11.375 in TCGA cohort, 6.02 in E-MTAB-6389 cohort and 5.255 in OEP001105 cohort, respectively. The unfavorable impact of COL12A1 upregulation on the prognosis of iCCA patients was also observed at the translational level in two independent iCCA cohorts (Fig. 3D, Additional file 2: Fig. S2), and the optimal cutoff point for stratifying iCCA patients into two groups was -0.6726799 (log2 ratio of unique peptides) in OEP001105 cohort and 3 (IHC score of COL12A1 staining) in LVC1202 cohort, respectively. The median overall survival time was significantly shorter in iCCA patients with COL12A1-high expression than iCCA patient with COL12A1-low expression from TCGA, OEP001105, E-MTAB-6389 and LVC1202 cohorts (18.55 (10.16–29.54) months vs. 53.45 (29.62–75.53) months, *p* = 0.014, Fig. 3E). Furthermore, the recurrence risk of iCCA was higher in COL12A1-high expression (COL12A1 staining score ≥ 4) group (Fig. 3F). Additionally, multivariate analyses determined that COL12A1-high expression was an independent risk factor associated with the poor prognosis of iCCA patients (Fig. 3G).

### COL12A1 promoter methylation pattern in iCCA and the corresponding nontumor samples

**Fig. 4:**
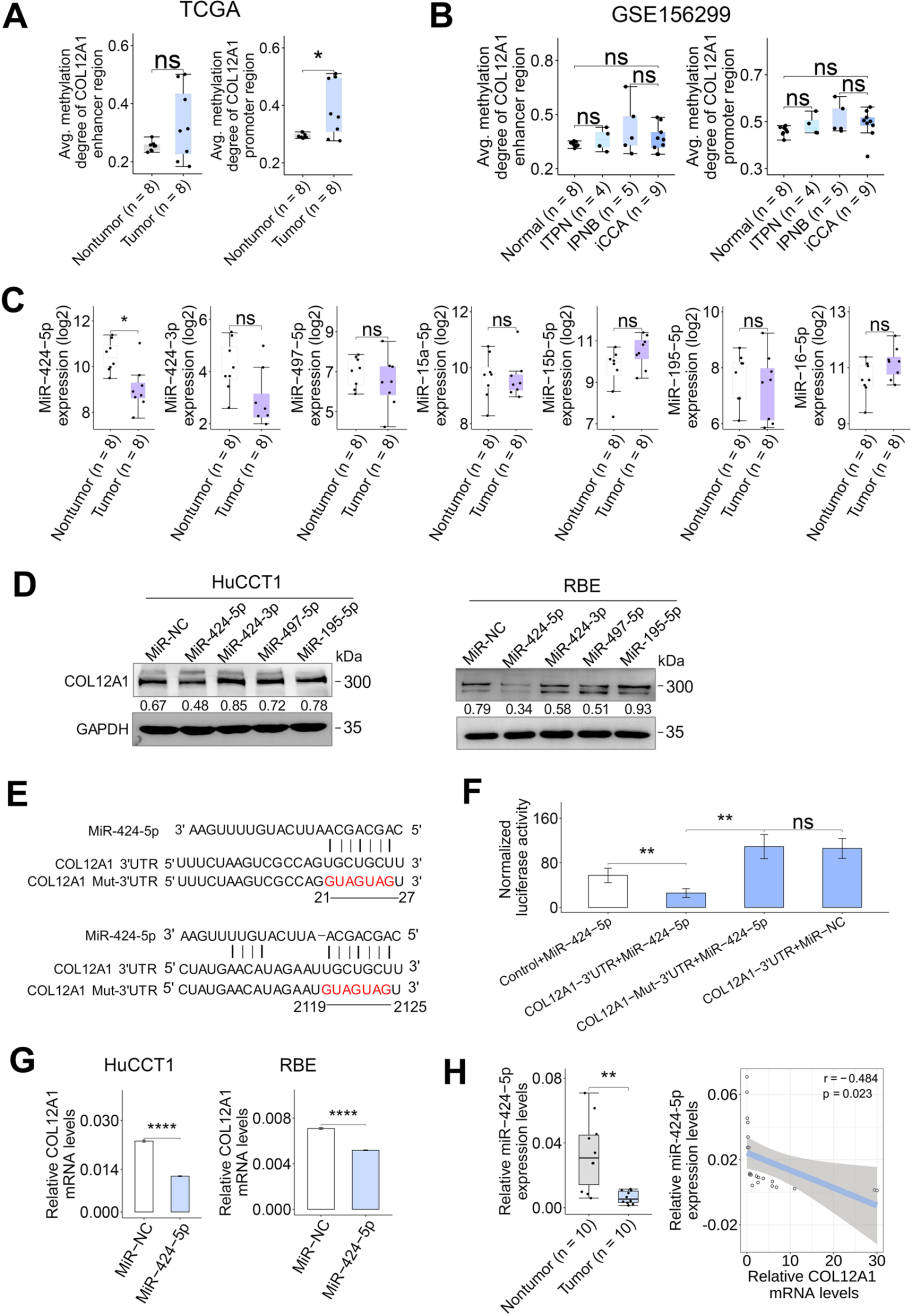
MiR-424-5p downregulation contributes to COL12A1 upregulation in clinical iCCA. **A** The methylation degree of COL12A1 enhancer/promoter region in iCCA and nontumor samples from TCGA. ns, *p* ≥ 0.05. *p < 0.05, using two-tailed paired Student's *t* test. **B** The methylation degree of COL12A1 enhancer/promoter region in iCCA, intraductal tubulopapillary neoplasm of biliary duct (ITPN), intraductal papillary neoplasm of biliary duct (IPNB) and nontumor samples from GSE156299. ns, *p* ≥ 0.05. *p < 0.05, using two-tailed unpaired Student's *t* test. **C** The expression patterns of candidate miRNAs (MiR-424-5p, -424-3p, -497-5p, -15a-5p, -15b-5p, -195-5p and miR-16-5p) in 8 pairs of iCCA samples from TCGA. ns, *p* ≥ 0.05. *p < 0.05, using two-tailed paired Student's *t* test. **D** COL12A1 in HuCCT1 and RBE cells after transfection of miR-NC, miR-424-5p, miR-424-3p, miR-497-5p, or miR-195-5p for 48 h was assessed by immunoblotting, respectively. **E** The putative binding sites of miR-424-5p in wild-type COL12A1 3'UTR aligned with vertical lines. The mutated bases are presented in red. **F** Luciferase report activity in HEK-293 T cells after co-transfected with COL12A1 3'UTR report and miR-424-5p mimic for 48 h compared to the control. ns, *p* ≥ 0.05. **p < 0.01, using two-tailed unpaired Student's *t* test. **G** COL12A1 mRNA levels in HuCCT1 or RBE cells after transfection of miR-NC or miR-424-5p for 48 h were assessed by RT-PCR, respectively. ****p < 0.0001, using two-tailed unpaired Student's *t* test. **H** MiR-424-5p expression level in 10 pairs of iCCA tissue samples was determined by RT-PCR. **p < 0.01, using two-tailed paired Student's *t* test. The relationship between COL12A1 mRNA and miR-424-5p in 10 pairs of iCCA samples was assessed by Pearson correlation test. Experiments were triplicated. *iCCA* intrahepatic cholangiocarcinoma, *RT-PCR* real-time PCR, *TCGA* The Cancer Genome Atlas

To investigate the upstream mechanism of COL12A1 upregulation in iCCA, we first deciphered the methylation pattern of COL12A1 promoter/enhancer regions by analysis of two independent DNA methylation datasets of clinical iCCA (Additional file 3: Fig. S3). The methylation degree of COL12A1 enhancer region was similar between iCCA tissues and the adjacent nontumor liver tissues in both TCGA and GSE156299 datasets (Fig. 4A, B); the methylation degree of COL12A1 promoter region was significantly increased in iCCA tissues relative to the paired nontumor liver tissues in TCGA dataset (Fig. 4A), while no significant difference was observed in the samples included in GSE156299 dataset (Fig. 4B). In addition, the methylation degree of several regions in COL12A1 locus including cg03564793, cg14375912, cg04074140 and cg19132213 was significantly increased in iCCA tissue samples compared with the adjacent nontumor samples (Additional file 4: Fig. S4), indicating that the methylation pattern of COL12A1 promoter is not the cause for its upregulation in human iCCA samples.

### MiR-424-5p directly binding to COL12A1 mRNA in iCCA cells

TargetScanHuman database (version 8.0) prediction revealed that 7 candidate miRNAs (miR-424-5p, -15a-5p, -497-5p, -15b-5p, -6838-5p, -16-5p and miR-195-5p) can bind to COL12A1 3'UTR (Additional file 5: Fig. S5). Both sample-paired and unpaired analyses determined that miR-424-5p expression was significantly decreased in clinical iCCA relative to the adjacent nontumor samples (Fig. 4C and Additional file 6: Fig. S6A). Except for miR-6838-5p that was not detected in TCGA dataset, the remaining candidate miRNAs expression levels were similar between clinical iCCA and the adjacent nontumor samples (Fig. 4C and Additional file 6: Fig. S6A). Correlation analyses showed that miR-424-5p was significantly negatively correlated with COL12A1 mRNA expression in clinical iCCA samples from TCGA (Additional file 6: Fig. S6B, C). Additionally, miR-424-5p was markedly downregulated in human iCCA cells compared with normal intrahepatic bile duct cell HIBEpiC (Additional file 6: Fig. S6D).

Immunoblotting showed that overexpression of miR-424-5p suppressed COL12A1 expression in HuCCT1 and RBE cells (Fig. 4D). Luciferase reporter assays showed that luciferase activity was markedly reduced or increased in HEK-293T cells co-transfected with miR-424-5p plus wild-type COL12A1 3'UTR or mutated 3'UTR of COL12A1, respectively (Fig. 4E, F). RT-PCR showed that overexpression of miR-424-5p downregulated COL12A1 mRNA expression in HuCCT1 and RBE cells (Fig. 4G). In addition, the downregulation of miR-424-5p in clinical iCCA and the inverse association of COL12A1 mRNA with miR-424-5p expression were validated in our internal iCCA dataset (Fig. 4H). These findings suggest that miR-424-5p can result in translational inhibition or degradation of COL12A1 by directly targeting COL12A1 mRNA.

### Promoter hypermethylation-mediated miR-424-5p downregulation in clinical iCCA

**Fig. 5:**
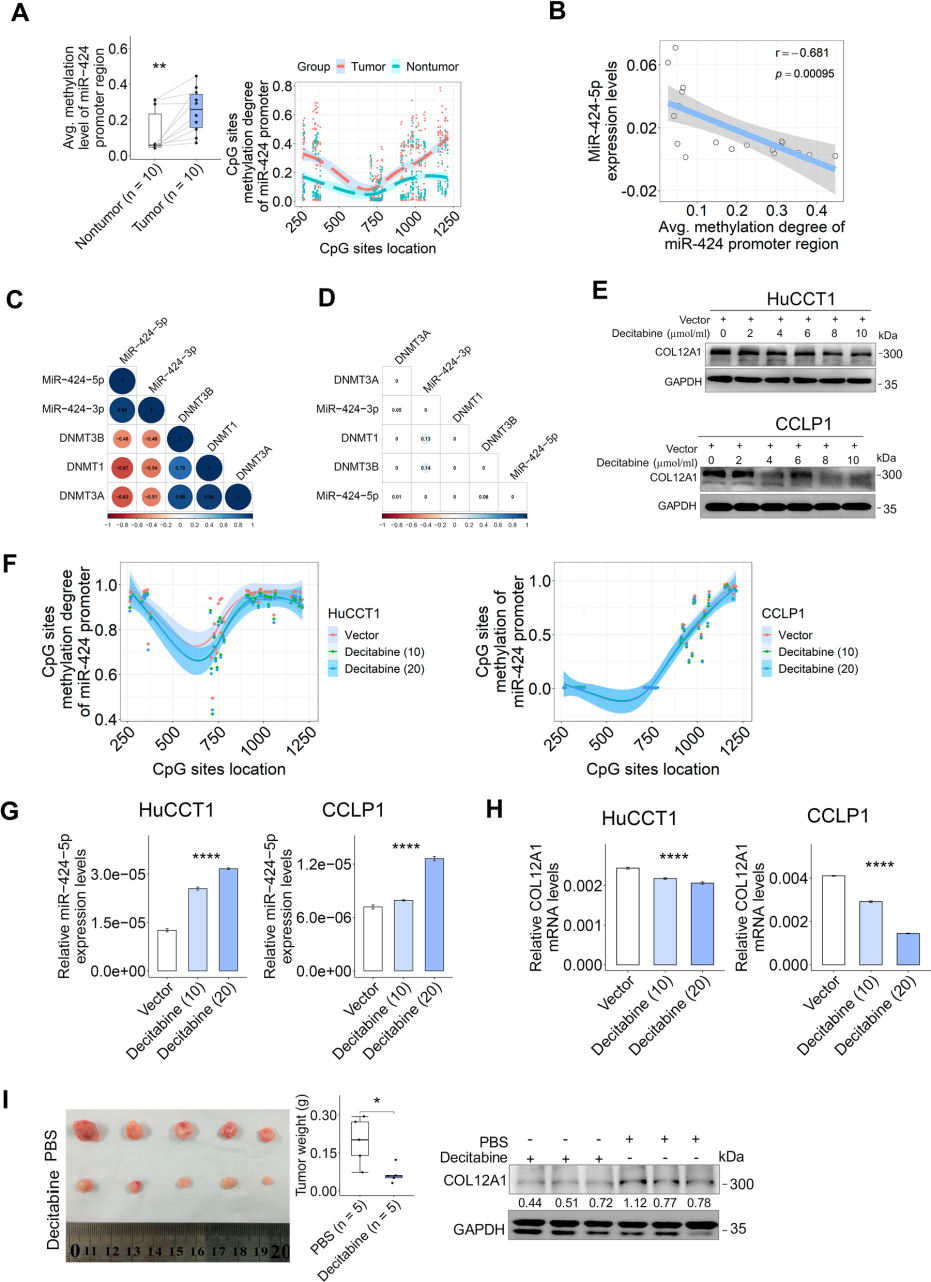
Promoter hypermethylation silenced miR-424-5p in clinical iCCA. **A** Promoter CpG sites methylation degree of miR-424 in 10 pairs of iCCA tissues samples. **p < 0.01, using two-tailed paired Student's *t* test. **B** The association of miR-424-5p with promoter methylation degree of miR-424 in 10 pairs of iCCA samples was assessed by Pearson correlation test. **C**, **D** Relationships between DNA methyltransferases (DNMTs) and miR-424 family members in 8 pairs of iCCA samples from TCGA were assessed by Pearson correlation test. Numbers in heatmaps denote Pearson coefficient (**C**) or statistical significance (**D**). **E** COL12A1 in the whole cell lysate of HuCCT1 or CCLP1 cells after treated with the indicated dose of decitabine for 48 h was evaluated by immunoblotting. **F** CpG sites methylation degree of miR-424 in HuCCT1 or CCLP1 cells after treated with 10/20 μmol/mL decitabine for 48 h was determined by targeted bisulfite sequencing. Decitabine was dissolved with phosphate-buffered saline (Vector). **G**, **H** MiR-424-5p and COL12A1 expression in HuCCT1 or CCLP1 cells after treated with 10/20 μmol/mL decitabine for 48 h was determined by RT-PCR, respectively. ****p < 0.0001, using one-way analysis of variance (ANOVA). **I** Decitabine attenuated the growth of subcutaneous xenograft tumors. NSG mice (*n* = 5 mice in each group) were treated with 2 mg/kg decitabine (5 times weekly) by tail vein injection. Tumor weights in the two groups were analyzed. COL12A1 in xenograft tumors from the indicated groups was assessed by immunoblotting. *p < 0.05, using two-tailed unpaired Student's *t* test. Experiments were in triplicates. *iCCA* intrahepatic cholangiocarcinoma, *RT-PCR* real-time PCR, *TCGA* The Cancer Genome Atlas

To decipher the upstream regulatory mechanism for miR-424-5p downregulation in clinical iCCA, we tested the promoter methylation pattern of miR-424 in 10 pairs of clinical iCCA samples by targeted bisulfite sequencing. The results showed that CpG sites methylation degree of miR-424 promoter area was markedly increased in iCCA compared with the corresponding paired nontumor samples (Fig. 5A). The promoter methylation degree of miR-424 was significantly inversely associated with miR-424-5p expression in iCCA (Fig. 5B). In addition, DNA methyltransferase writers (DNMTs) were inversely correlated with miR-424 family members at the transcriptional level (Fig. 5C, D). These results indicate that DNMTs-mediated promoter CpG sites hypermethylation results in miR-424-5p downregulation in clinical iCCA.

We treated iCCA cells with decitabine to assess whether DNA demethylating agents can inhibit COL12A1 expression via inducing miR-424 promoter demethylation. Immunoblotting showed that COL12A1 expression was decreased in HuCCT1 and CCLP1 cells after being treated with the indicated dose of decitabine (Fig. 5E). Targeted bisulfite sequencing showed that CpG sites methylation degree of miR-424 promoter area was decreased after decitabine treatment in HuCCT1 and CCLP1 cells (Fig. 5F). RT-PCR determined that miR-424-5p expression was increased whereas COL12A1 mRNA expression was decreased in HuCCT1 and CCLP1 cells treated with decitabine (Fig. 5G, H). Moreover, COL12A1 expression levels were lower in subcutaneous xenograft tumors from the decitabine treatment group compared with the controls (Fig. 5I). Collectively, these findings showing that DNMTs-mediated miR-424-5p downregulation resulted in COL12A1 upregulation in iCCA cells.

### COL12A1 promoting proliferation and growth of iCCA cells in vitro and in vivo

**Fig. 6:**
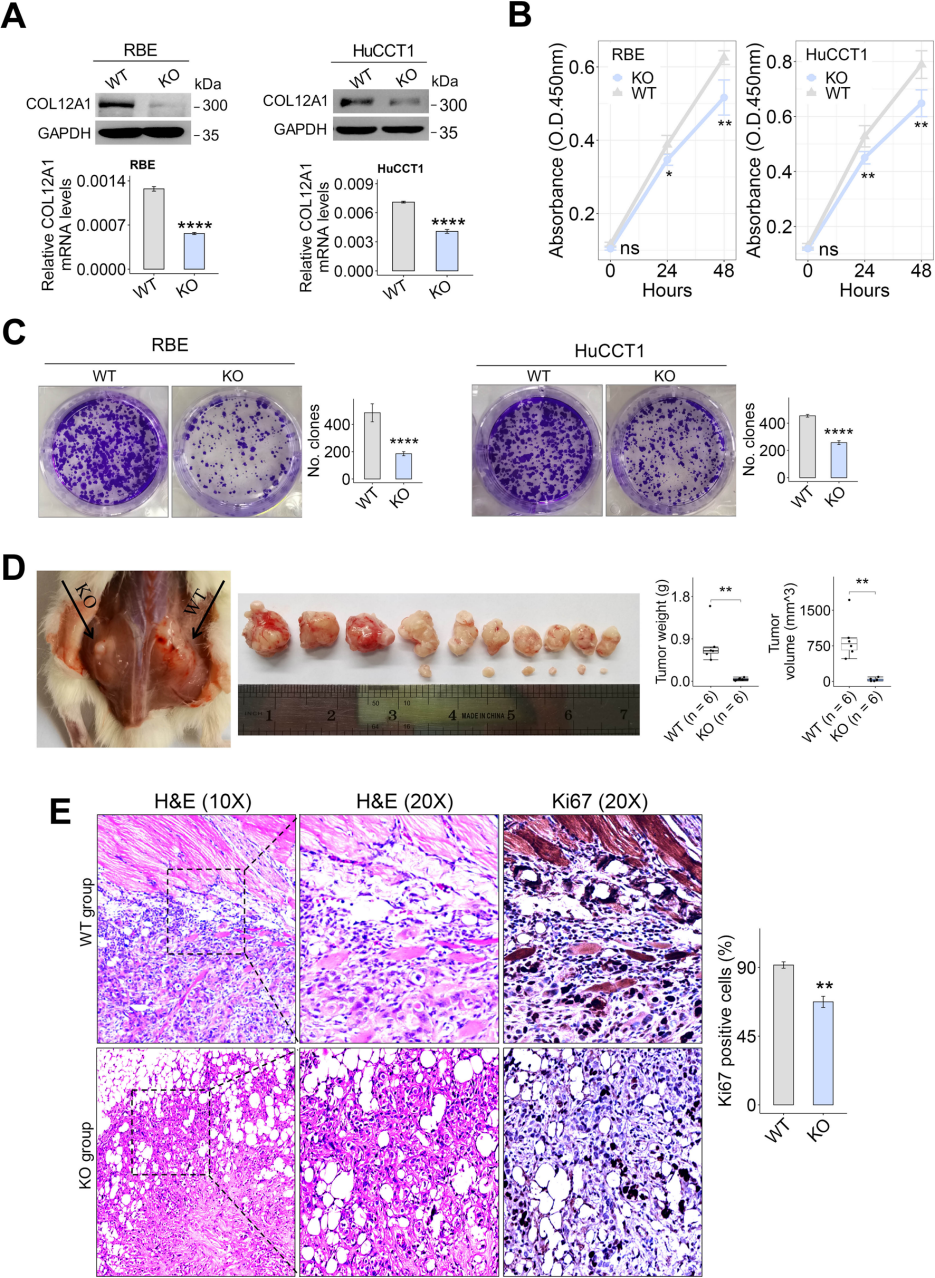
COL12A1 promotes iCCA cell growth. **A** CRISPR/Cas9-mediated COL12A1 knockout efficiency in HuCCT1 or RBE cells was evaluated by immunoblotting and RT-PCR, respectively. **B** The impact of COL12A1 knockout on HuCCT1 or RBE cells proliferation was evaluated by CCK8 assay. **C** Representative images of colony-forming assays and the colony numbers in HuCCT1 or RBE cells with or without COL12A1 knockout. **D** Representative gross images of subcutaneous xenograft tumor with or without COL12A1 knockout. Xenograft tumor volumes and weights were analyzed between the two groups (*n* = 10 mice). **E** Representative images of H&E and Ki67 staining of subcutaneous xenograft tumor. Original magnification: 10/20X. Bar plot to show Ki67 positive cells from 5 different fields in xenograft tumor slides of the two groups. Experiments were in triplicates. *p < 0.05, **p < 0.01, ****p < 0.0001, using two-tailed unpaired Student's *t* test. *iCCA* intrahepatic cholangiocarcinoma

To assess the impact of COL12A1 on iCCA cell progression, we silenced COL12A1 expression in HuCCT1 and RBE cells using COL12A1 CRISPR/Cas9 KO plasmid, respectively (Fig. 6A). In cell viability assays, COL12A1 knockout weakened HuCCT1 and RBE cells proliferation (Fig. 6B). In colony-forming assays, COL12A1 knockout inhibited tumor cells growth by attenuating colony-forming ability of iCCA cells (Fig. 6C). In subcutaneous xenograft models, COL12A1 knockout inhibited tumor growth and proliferation (Fig. 6D, E).

### COL12A1 positively correlated with the epithelial–mesenchymal transition (EMT) program in clinical iCCA

**Fig. 7:**
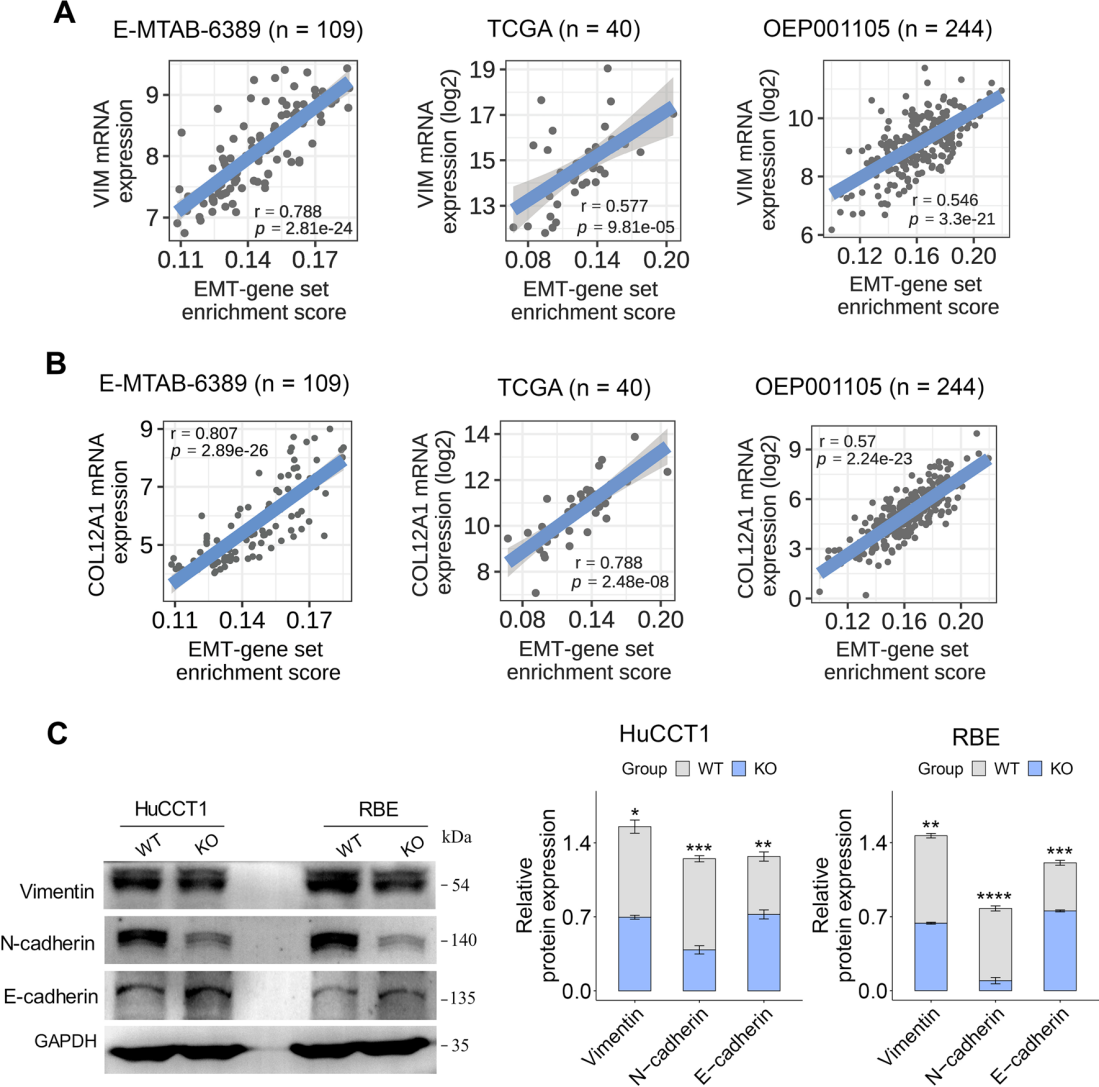
COL12A1 regulates the EMT program of iCCA cells. **A**, **B** Scatter plot showing the relationship between vimentin (VIM)/COL12A1 mRNA expression and the EMT-gene set enrichment score in three iCCA transcriptomic datasets (including E-MTAB-6389, TCGA and OEP001105). *p* value is based on Pearson correlation test. **C** The EMT-associated proteins in whole cell lysate of HuCCT1 and RBE cells with or without COL12A1 knockout were evaluated by immunoblotting. Bar plots showing the relative expression level of EMT-associated proteins in the two groups. Experiments were in triplicates. *p < 0.05; **p < 0.01; ***p < 0.001; ****p < 0.0001, based on two-tailed unpaired Student's *t* test. *iCCA* intrahepatic cholangiocarcinoma, *EMT* epithelial–mesenchymal transition

Like the EMT-associated marker vimentin (VIM), COL12A1 was significantly positively correlated with EMT-gene set enrichment score in 3 independent iCCA datasets (Fig. 7A, B). Immunoblotting showed that COL12A1 knockout increased the epithelial marker E-cadherin expression whereas reduced mesenchymal markers (N-cadherin and vimentin) expression in HuCCT1 and RBE cells (Fig. 7C). These findings indicate that COL12A1 upregulation plays a key role in maintaining the EMT program of iCCA cells.

### The therapeutic value of miR-424-5p in iCCA by sponging COL12A1

**Fig. 8:**
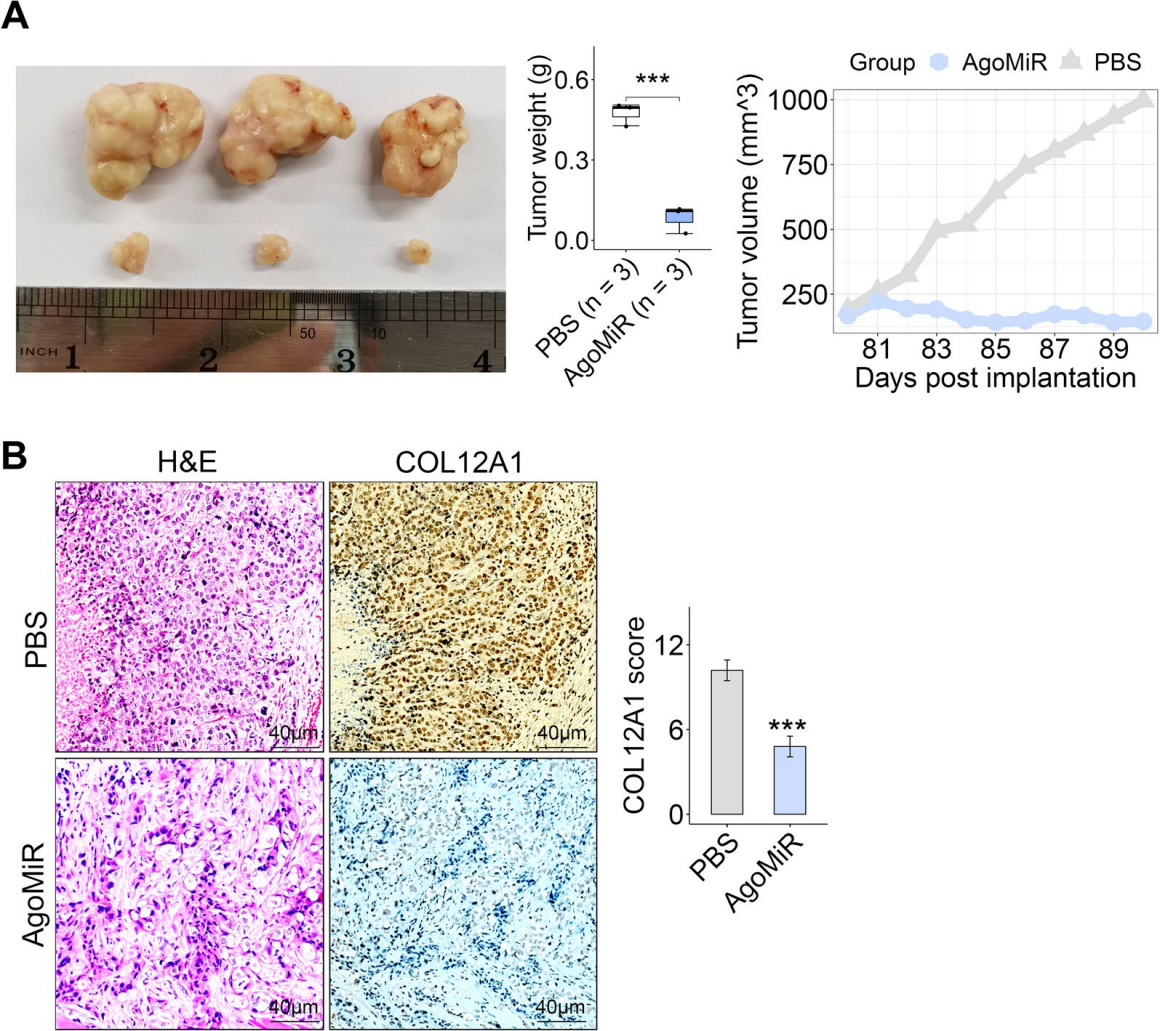
Administration of miR-424-5p agonist inhibited xenograft tumor growth. **A** Tumor weight and volume were decreased in AgomiR (MiR-424-5p agonist) group compared with the controls (PBS). **B** Representative image of H&E and COL12A1 staining in subcutaneous xenograft tumor from AgomiR or control groups. Boxplot showing COL12A1 expression levels in the two groups. ***p < 0.001, using two-tailed Student’s *t* test.

MiR-424-5p overexpression can inhibit the growth, invasion and migration of iCCA cell in vitro [12]. However, it is still unclear whether miR-424-5p has antitumor potential in vivo. To evaluate the treatment effect of miR-424-5p in iCCA, we treated the tumor-bearing mice by tail vein administration of miR-424-5p agonist (AgomiR, 20 nmol per mouse). In the xenograft model, tumor growth was markedly inhibited in AgomiR treatment group relative to the controls (Fig. 8A). COL12A1 expression was decreased in the AgomiR group compared with the controls (Fig. 8B).

## Discussion

In the present study, we identified 1669 potential iCCA-specific biomarkers by reanalysis of multiple transcriptomic datasets of iCCA and HCC. These results will contribute to more precisely revealing tumor-specific molecular signatures involved in iCCA initiation and/or progression and discovering potential therapeutic targets for iCCA patients. The omics data provided valuable resources for determining the common and distinct events at genomic, epigenetic, transcriptomic and proteomic scale in iCCA [6–9, 11, 13, 14]. By integrative analysis of transcriptomics and clinical profiles of iCCA patients, we are interested in aberrantly expressed genes clustered in gene expression module correlated with necrosis that occurred in tumor and overall survival of iCCA patients. Based on the results from enrichment analyses and the marked collagen-rich tumor microenvironment in iCCA, we subsequently investigated the role of COL12A1 in iCCA.

COL12A1 is aberrantly upregulated in human malignancies including gastric cancer, colon cancer, colorectal cancer and pancreatic adenocarcinoma, and its upregulation correlated with tumor progression and poor prognosis [15–18]. Consistent with previous findings, the present research showed that COL12A1 is significantly upregulated in clinical iCCA relative to the nontumor liver tissues or HCC samples. Higher expression of COL12A1 was associated with tumor progression and worse survival in iCCA patients. Moreover, our loss-of-function experiments demonstrated that COL12A1 upregulation promotes iCCA cells proliferation and growth in vitro and in vivo. Thus, COL12A1 is a promising biomarker for the prognosis prediction of iCCA patients.

EMT program plays a pivotal role in the growth and metastasis of cancers including iCCA [5, 19]. In the present study, we first revealed that, like vimentin (a classic mesenchymal marker), COL12A1 is positively correlated with EMT-gene set enrichment scores in clinical iCCA samples, indicating that COL12A1 mRNA may be served as a new EMT maker for clinical iCCA. Additionally, we found that COL12A1 can regulate EMT biomarkers expression at the protein level in human iCCA cells, which suggests that COL12A1 regulates the growth and metastasis process of iCCA cells possibly via modulating the EMT program.

Accumulating evidence suggests that epigenetic dysregulation modulates liver cancer development by regulating tumor-associated gene expression [20–23]. Unlike promoter/enhancer demethylation-mediated upregulation of ITPR3, MCM2 and NUP37 in hepatocellular carcinoma [20, 21]. In this study, we first revealed that DNA methylation degree of COL12A1 promoter/enhancer was not demethylated in iCCA relative to the adjacent nontumor samples, indicating that COL12A1 upregulation is independent of the methylation level of its promoter/enhancer region in clinical iCCA.

MicroRNA dysregulation plays a pivotal role in human diseases, particularly in cancers [12, 24]. In the present study, we first revealed that miR-424-5p is an upstream regulator of COL12A1 in iCCA. It has been demonstrated that miR-424-5p was downregulated in several kinds of cancer and had antitumor functions in breast cancer and iCCA cells [12, 25]. Consistent with the previous finding, our results also showed that miR-424-5p was markedly downregulated in clinical iCCA tissues relative to the paired nontumor liver tissues.

MiR-424-5p can regulate Hippo/YAP1 and Wnt/ꞵ-catenin signaling via targeting circACTN4 and YAP1 in iCCA cells [12], showing that miR-424-5p plays a crucial role in iCCA initiation and/or progression and may have therapeutic potential via inhibiting Hippo/YAP1 and Wnt/ꞵ-catenin signaling in iCCA. In our study, we observed that miR-424-5p had therapeutic potential in the xenograft iCCA model via targeting COL12A1. These results elucidated the pivotal role of miR-424-5p in iCCA progression and provided preclinical evidence for further translational studies to investigate the therapeutic effect of miR-424-5p in iCCA patients.

There is still a lack of research to decipher the epigenetic regulatory mechanism of miR-424-5p downregulation in human malignancies. In this study, we first demonstrated that promoter CpG sites of miR-424 were aberrantly hypermethylated in iCCA, which induced miR-424-5p downregulation in clinical iCCA. Furthermore, DNMTs-dictated aberrant promoter hypermethylation silenced miR-424-5p in iCCA cells. Although we first revealed the epigenetic mechanism of COL12A1 upregulation in clinical iCCA and determined the protumor function of COL12A1 in human iCCA cells, there are also some limitations in our present study. First, we only established HuCCT1 cell-derived xenograft mouse models to assess the role of COL12A1 in vivo, there may be heterogeneities among different iCCA models such as patient-derived xenograft and hydrodynamic model of iCCA. Second, we did not evaluate the therapeutic effect of miR-424-5p mimic in iCCA patients via targeting COL12A1 since there is still a lack of public evidence to make a clinical trial. Future studies are needed to explore the detailed mechanisms downstream of COL12A1 in iCCA progression and develop potential inhibitor-targeting COL12A1 mRNA or protein for in vivo use.

## Conclusions

COL12A1 is upregulated in iCCA by promoter CpG sites hypermethylation-induced downregulation of miR-424-5p, which promotes iCCA progression. MiR-424-5p has therapeutic potential in iCCA by targeting COL12A1. Our research determined the pivotal role of COL12A1 in iCCA progression and indicated that COL12A1 is a promising druggable target for epigenetic therapy of iCCA. Therefore, it is reasonable for use of miR-424-5p mimic or potential inhibitor-targeting COL12A1 to manage iCCA progression or postoperative recurrence. This hypothesis should be tested in future translational studies enrolling iCCA patients.

## Methods

### Public omics datasets

Public bulk-RNA seq datasets (TCGA-CHOL [6], TCGA-LIHC [26], GSE76297 [8], GSE26566 [27], E-MTAB-6389 [9], GSE32879 [28], E-MTAB-4171 [29], OEP001105 [11] and OEP000321 [30]), including gene expression profile of 565 intrahepatic cholangiocarcinoma (iCCA), 607 hepatocellular carcinoma (HCC), 7 combined hepatocellular carcinoma (CHC), 5 focal nodular hyperplasia (FNH) and 327 adjacent nontumor liver tissue specimens included in The Cancer Genome Atlas (TCGA), ArrayExpress, Gene Expression Omnibus (GEO), and National Omics Data Encyclopedia (NODE) were obtained and processed using R package TCGAbiolinks (version 2.20.1), oligo (version 1.56.0) and GEOquery (version 2.60.0), respectively [31–33]. Differentially expressed genes between nontumor liver tissue and iCCA or HCC samples were inferred using R package limma (version 3.50.3) based on |logFC| > 0.1 and *p* value < 0.05 [34].

Re-analyses of DNA methylation datasets GSE156299 [35] and TCGA-CHOL [6] were performed to decipher the methylation patterns of COL12A1 gene in iCCA and nontumor liver tissues. The raw data of DNA methylation were processed using minifi (version 1.38.0) as previously described [21, 36]. Weighted gene co-expression network analysis (WGCNA) was performed for weighted correlation network analysis of gene expression patterns in iCCA [37].

Single-sample gene set enrichment was performed using R package GSVA (version1.42.0) and GSEABase (version 1.56.0), and epithelia-to-mesenchymal transition (EMT) gene sets (GOBP_EPITHELIAL_TO_MESENCHYMAL_TRANSITION) in Molecular Signatures Database c5.all.v7.5.1.symbols.gmt was applied to calculate the EMT-gene set enrichment score in each sample [38].

### Patients and clinical tissue specimen

A total of 10 iCCA, 10 HCC, and the paired nontumor liver tissue samples were prospectively collected from treatment-naive patients with iCCA or HCC post radical surgical treatment from May to September 2020 at our hospital. The clinicopathological characteristics of 10 iCCA/HCC patients are summarized in Additional file 8: Table S2. After snap-frozen in liquid nitrogen, the collected tissue samples were stored at − 80 refrigerator for subsequent molecular biology experiments as we previously described [21]. An external cohort (LVC1202, SUPERBIOTEK, Shanghai, CN), including 60 primary iCCA patients without receiving neoadjuvant treatment prior to primary surgery, was obtained for survival analysis. The TNM staging of each iCCA patient after surgical resection was performed in accordance with the 8th edition of the American Joint Committee on Cancer (AJCC) TNM classification system [39]. The pathological diagnosis of iCCA or HCC was performed according to World Health Organization criteria [40]. Written informed consent was collected from each patient enrolled in the present study. The experimental protocol (5826Z/20, 0531B/22) for using clinical tissue specimens was reviewed and approved by Institutional Ethics Committee in our hospital.

### Cell lines, cell culture, stable cell line establishment and transient infection

RBE cell line was obtained from Yuchi Biological Technology Co., Ltd. (Shanghai, CN). HuCCT1 (JCRB0425) and HCCC9810 cell lines were kindly provided by the Key Laboratory of Carcinogenesis and Cancer Invasion (Fudan University), Ministry of Education, Shanghai, CN. CCLP1 and normal human intrahepatic biliary cell (HIBEpiC) lines were kindly provided by Key Laboratory of Combined Multiorgan Transplantation, National Health Commission, Hangzhou, Zhejiang, CN. HEK-293T cell line was purchased from the Cell Bank of Chinese Academy of Sciences (Shanghai, CN). RBE cell line came from a 37-year-old yellow race woman with primary iCCA. HCCC9810 cell line came from a 60-year-old yellow race woman with primary iCCA. CCLP1 cell line came from a 48-year-old Caucasian female with primary iCCA. HuCCT1 cell line came from a 56-year-old yellow race man with primary iCCA. HIBEpiC cells were isolated from normal liver tissue. All cell lines were tested by short tandem repeat typing. Furthermore, all cells were cultured in RPMI-1640 medium (Thermo Fisher Scientific, USA) containing 10% fetal bovine serum (FBS) (Thermo Fisher Scientific, USA), 100 U/mL penicillin (Cienry, Huzhou, CN) and 100 μg/mL streptomycin (Cienry, Huzhou, CN) at 37 °C in 5% CO2 atmosphere. Human hepatoma cell lines (including Hep3B, Bel-7402, HCC-LM3 and Huh7) and normal liver cell line HL7702 were cultured as we previously described [21]. Additionally, all of the cell lines had been tested regularly for mycoplasma contamination as previously described [21].

COL12A1 CRISPR/Cas9 KO plasmid (sc-402361) was purchased from Santa Cruz Biotechnology (USA). 2 × 10^5^ HuCCT1 or RBE cells (per well) in the logarithmic phase were seeded in a 6-well tissue culture plate and incubated with antibiotic-free standard growth medium (3 mL per well). Upon cells growing to a 50–70% confluency after initial seeding, 2 μg (0.1 μg/μL) of plasmid DNA diluted in 130 μL of Plasmid Transfection Medium (sc-108062, Santa Cruz Biotechnology, USA) and 10 μL of UltraCruz® Transfection Reagent (sc-395739, Santa Cruz Biotechnology, USA) diluted in 140 μL of Plasmid Transfection Medium, were transfected into HuCCT1 and RBE cells following the manufacturer’s protocol. After incubation for 48 h, cells with successful transfection of COL12A1 KO plasmid were determined by detecting the green fluorescent protein (GFP) using fluorescent microscopy (Leica DMi8, Germany). Then, after cell sorting for GFP by FACS (MoFloAstrios EQ, Beckman), the harvested cells were cultured in a standard growth medium for 3–4 weeks and the selected single-cell colony with COL12A1 knockout was then used for the downstream experiments.

The mimic of miRNAs including miR-424-5p (miR10001341-1-5), miR-424-3p (miR10004749-1-5), miR-497-5p (miR10002820-1-5), miR-195-5p (miR10000461-1-5) and the negative control (miR-NC, miR1N0000001-1-10) were purchased from RIBOBIO Co. Ltd. (Guangzhou, CN). Transient transfection with the mimic of miRNAs was performed using riboFECT CP Transfection Kit (C10511-05, RiboBio Co. Ltd., Guangzhou, CN) in accordance with the manufacturer's protocol.

DNA methylation inhibitor decitabine (S1200) was purchased from Selleck (Shanghai, CN). 3 × 10^5^ HuCCT1 or CCLP1 cells (per well) were seeded in a 6-well cell culture plate and incubated for 48 h in RPMI-1640 containing 10% FBS and 10/20 μmol/mL decitabine.

### Protein extraction and immunoblotting

Protein extraction from fresh clinical tissue samples or human cell lines and immunoblotting procedures were performed as previously described [21]. Samples containing 20–40 μg proteins were subjected to SDS-PAGE (8–10% polyacrylamide) and blotted on polyvinylidene difluoride (PVDF) membranes. After blocking with 5% skim milk, the membranes were first incubated with COL12A1 antibody (1:2000, Sigma-Aldrich, #HPA009143), Vimentin antibody (1:1000, Abcam, #ab92547), E-cadherin antibody (1:1000, Cell Signaling Technology, #3195), or N-cadherin antibody (1:1000, Cell Signaling Technology, #13116), and then incubated with Goat Anti-Rabbit IgG H&L (1:5000, Abcam, #ab205718). GAPDH (1:5000, Abcam, #ab181602) was used as a loading control.

### RNA isolation and real-time PCR

Total RNA isolation from clinical tissue samples or cell lines was performed using TRIzol™ Plus RNA Purification Kit (12183555, Invitrogen™, Thermo Fisher Scientific, USA) following the instruction for the user. Real-time PCR was performed as previously described [21]. The primers of the human COL12A1 and β-actin gene (GCD0259363) were purchased from GeneChem (Shanghai, CN). The primer sequences of COL12A1 and β-actin are as follows: COL12A1, forward 5′-AGGTCGGATGACGGGAAGA-3′ and reverse 5′-GCGGACATTCAAGGTGCTG-3′; β-actin, forward 5′-CGACAGATGCAGAACGAGA-3′ and reverse 5′-GACCCTGGATGTGACAGCTC-3′.

MicroRNAs (miRNAs) in clinical tissue samples and human cell lines were extracted using an ultrapure miRNA isolation kit (5080576001, Roche) following the instructions for the user. The cDNA template was produced using Bulge-Loop miRNA qRT-PCR Starter Kit (C10211-2, RIBOBIO, Guangzhou, CN) following the instructions for the user. U6 snRNA was used as the endogenous control, and miRNAs primers (MQPS0001272-1-200, MQPS0001271-1-200, MQPS0000002-1-200) were purchased from RiboBio Co. Ltd. (Guangzhou, CN). A final volume of 20 μL mixture containing Bulge-Loop miRNA qRT-PCR Starter Kit (C10211-1, RIBOBIO, Guangzhou, CN) reagents, RT-PCR Grade Water (AM9935, Applied Biosystems, Thermo Fisher Scientific, USA) and cDNA template was amplified using ABI QuantStudio-5 Real-Time PCR System (Applied Biosystems, Thermo Fisher Scientific, USA) in accordance with the manual for users. Reaction conditions were set as 95 °C for 10 min, 40 cycles of denaturation at 95 °C for 2 s, annealing at 60 °C for 20 s and extension at 70 °C for 10 s. The ΔΔCT method was used to determine the relative miRNA expression level in each sample.

### Hematoxylin and eosin staining and immunohistochemistry

Immunohistochemical (IHC) or Hematoxylin and eosin (H&E) staining was performed as previously described [2, 21]. Paraffin-embedded liver tissue sections (3 μm thickness) were incubated with anti-COL12A1 (1:200, Sigma #HPA009143) or anti-Ki67 (1:300, Abcam, #ab16667), respectively. The COL12A1 staining score was used to divide iCCA patients into COL12A1-low and COL12A1-high expression groups based on an optimal threshold.

### Immunofluorescence

A total of 2 × 10^3^ iCCA cells, seeded on coverslips coated in a 24-well cell culture plate, were incubated in RPMI-1640 medium containing 10% FBS and allowed to adhere for 6–8 h. After 3 times washing using phosphate-buffered saline (PBS), cells were fixed with 4% paraformaldehyde for 10 min at room temperature, then washed 3 times with ice-cold PBS, permeabilized with 0.1% Triton-X100 in PBS for 10 min, blocked with 1% bovine serum albumin (BSA) and 22.52 mg/mL glycine in PBS supplemented with 0.1% Tween 20 (PBST) at room temperature for 30 min, and finally incubated with anti-COL12A1 antibody (1:50) overnight at 4 °C in the dark. After 3 times washing using PBS, cells were then incubated with fluorescence-labeled secondary antibody (1:200, Abcam, #ab150079) for 1 h in the dark, washed 3 times with PBS, followed by incubation with 0.1 μg/mL 4′,6-diamidino-2-phenylindole (DAPI) at room temperature for 1 min in the dark, and finally rinsed 3 times with PBS in the dark. Coverslips were mounted with UltraCruz® Aqueous Mounting Medium (sc-24941, Santa Cruz) and sealed with nail oil. Finally, cells on the coverslip were microphotographed using a Leica fluorescence microscope (TCS SP8, Germany).

### Cell viability assay

We assessed iCCA cell viability using CCK8 assays (Beyotime, Shanghai, CN) as previously described [21]. Briefly, 1 × 10^3^ iCCA cells (per well) were seeded into 96-well plates and incubated for 24 h and 48 h, respectively. Then, CCK8 (1:10) combined with fresh growth medium was added to each well. After incubation for 1 h, the absorbance at 450 nm was tested as previously described [21].

### Cell colony-forming assay

1 × 10^3^ iCCA cells in the logarithmic phase were seeded into a 6-cm cell culture dish and incubated for 2 weeks. After 3 times washing with PBS, the cell colonies were stained with 0.05% crystal violet (Sigma-Aldrich, USA) for 30 min, then photographed and counted using ImageJ (version.1.8.0), which were performed at room temperature.

### Dual-luciferase reporter assays

The sense and antisense fragments of the human COL12A1 3'UTR including either the wild-type (GOSE0310921) or the mutated sites (GOSE0310922) binding to miR-424-5p were synthesized separately by GeneChem (Shanghai, CN). Then the respective sense and corresponding antisense strands were hybridized and then cloned into the GV272-Firefly-Luciferase vector at the XhoI and KpnI sites. 2 × 10^4^ HEK-293 T cells (per well) were seeded into a 96-well cell culture plate, and co-transfected with either the GV272 vector (Control), GV272-COL12A1-3'UTR (wild-type), or the GV272-COL12A1-Mut-3'UTR, and the miR-424-5p mimic and CV045-TK promoter-Renilla-Luciferase plasmid (GeneChem, Shanghai, CN) using Lipofectamine^TM^3000 transfection reagent (Invitrogen, Thermo Fisher Scientific, USA) following the instructions for the user. After incubation for 48 h at 37 °C in 5% CO2, the luciferase activity of cell lysate was tested using a Dual-Glo luciferase assay kit (E1910, Promega). Luminescence was measured by a SpectraMax i3x Multi-model Microplate reader and the SoftMax Pro7 (Molecular Devices, USA).

### Genomic DNA isolation and targeted bisulfite sequencing

The extraction of genomic DNA in fresh-frozen iCCA and the paired nontumor liver tissue samples (*n* = 10), as well as human iCCA cells, was performed as we previously described [21]. The concentration of each extracted DNA sample was fluorometrically tested using Qubit 4.0 (Invitrogen, Thermo Fisher Scientific). Bisulfite conversion and next-generation sequencing (NGS) library preparation were performed as previously described [41]. According to the miRNA promoter recognition methods described by Annalisa Marsico et al. [42], we identified miR-424 promoter locus (chrX: 133683002–133684302) and designed primers for miR-424 promoter region using MethPrimer (version 2.0.0) [43, 44]. The putative primers for miR-424 promoter region (Additional file 9: Table S3) were validated and synthesized by BGI genomics (Shenzhen, CN). A total of 1 µg genomic DNA was subjected to bisulfite DNA conversion with ZYMO EZ DNA Methylation-Gold Kit (#D5006, Zymo Research, Irvine, CA, USA) accordingly to the manufacturer’s instructions. Then, a total of 500 ng bisulfite-converted DNA sample was used for PCR amplification using KAPA HiFi HotStart Uracil^+^ ReadyMix PCR Kit (#KK2801, Kapa Biosystems, Wilmington, MA, USA) combined with bisulfite-specific primers according to the standard conditions.

After the quality control using Labchip GX Touch (PerkinElmer, Germany), one-twentieth of the elution PCR reaction products were subjected to second round PCR for ligation of barcoded sequencing adapter with T4 DNA ligase. Thereafter, all samples were quantified on an Agilent 2100 (Agilent, USA) and adjusted for the same copy number for subsequent sequencing, and the sequencing was performed on a NovaSeq6000 system (Illumina, USA) using the NovaSeq 6000 S4 Reagent Kit v1.5 (300 cycles) in collaboration with Yunbios (Shanghai, CN).

The basic statistics on the quality of the raw reads was performed using The FastQC tool (http://www.bioinformatics.babraham.ac.uk/projects/fastq). The adapters and low-quality reads were removed using Trimmomatic v0.36 with default parameter [44]. The alignment of the bisulfite sequence to the human reference genome (hg19) was performed using the BSMAP v2.7.3 with parameters ‘-n 0 -g 0 -v 0.08 -m 50 -× 1000’ [45]. The unique mapped reads were kept for the subsequent analyses and the sequence depths of methylated cytosines were greater than 20X.

### Experimental animal model

The animal experiments were performed as previously described [21]. The experimental protocol (1615/22) for all animal experiments was reviewed and approved by the Institutional Animal Care & Use Committee (IACUC). Five-week-old male NSG mice used in the study were purchased from Model Animal Research Centre of Nanjing University, Nanjing, CN.

To determine the direct impact of COL12A1 on iCCA progression, 4 × 10^6^ HuCCT1 cells with or without COL12A1 knockout, resuspended in 200 μL PBS, were subcutaneously implanted into the flanks of 5-week-old male NSG mice. After observations for 8 weeks for tumor formation, mice were killed according to IACUC protocol. The collected tumor lesions were measured and weighted by a digital caliper and an electronic balance, respectively, and then fixed tumor tissue with 4% paraformaldehyde for use in downstream experiments. The tumor volume was calculated based on the formula as previously described [21].

Decitabine drug treatment model: HuCCT1 cells (3 × 10^6^) were implanted into the flanks of 5-week-old male NSG mice. Upon tumor reaching 25 to 50 mm^3^, mice were randomly divided into two groups with each group including 5 mice. Then, mice were treated with decitabine (2 mg/kg, 5 times weekly) or PBS by tail vein injection.

MiR-424-5p agonist (modified miR-424-5p mimic) administration model: After implantation of 3 × 10^6^ HuCCT1 cells into the flanks of 5-week-old male NSG mice, tumor development was monitored 3 times weekly at 3wks after implantation. Upon tumor reaching 130–200 mm^3^, mice were randomly divided into AgoMIR (20 nmol, i.v. 2 times weekly) group or control (100ul PBS, i.v. 2 times weekly) group, with each group consisting of 3 mice. AgoMIR (miR40001341-4-5) was purchased from RIBOBIO Co. Ltd. (Guangzhou, CN).

### Statistical analysis

All statistical analyses were performed with R v.4.1.0 (R Foundation for Statistical Computing, Vienna, Austria) or with SPSS v.22.0 (IBM Corp., Armonk, NY, USA). Unless otherwise indicated, data are expressed as means ± standard error of the mean (SEM). A comparison of two groups was made using a two-tailed Student’s *t* test or Wilcoxon test. A comparison of multiple groups was made using a one-way analysis of variance (ANOVA). Correlation analysis between two variables was assessed by Pearson correlation analysis. The association between COL12A1 expression and the clinicopathological features of iCCA patients was assessed by chi-square test or Fisher's exact test. Survival analysis was performed with the Kaplan–Meier method, and the statistical difference was determined by log-rank test. The “surv_cutpoint ()” function in R package survminer (https://cran.r-project.org/web/packages/survminer/index.html) was applied to determine the optimal cutoff point of COL12A1 mRNA/protein expression level corresponding to the survival of iCCA patients. Cox proportional hazards regression model was applied for multivariate survival analysis.

## Supplementary Information

**Additional file 1: Fig. S1.** COL12A1 mRNA expression is upregulated in clinical iCCA. (**A**) Boxplot to show COL12A1 mRNA expression levels in clinical iCCA, CHC, FNH and adjacent nontumor tissues samples from GSE32879 dataset. ns, *p* ≥ 0.05, **** *p* < 0.00001, using two-tailed unpaired Student's *t* test. Boxplot to show COL12A1 mRNA expression levels in clinical iCCA and HCC tissue samples form TCGA, GSE76297 or our in-house dataset, respectively. * *p* < 0.05; **** *p* < 0.00001, using two-tailed paired/unpaired Student's *t* test accordingly. Gallbladder or extrahepatic carcinoma tissue samples (*n* = 4) in TCGA cholangiocarcinoma dataset were excluded from our analysis. (**B**) Boxplot to show COL12A1 mRNA expression levels in clinical HCC and nontumor liver tissue samples from TCGA, E-MTAB-4171, GSE76297 or OEP000321 dataset, respectively. ns, *p* ≥ 0.05, using two-tailed paired/unpaired Student's *t* test accordingly. (**C**) COL12A1 protein levels in whole cell lysate of human HCC cell lines (including Hep3B, Bel-7402, HCC-LM3, and Huh7) and normal liver cell line HL7702 were evaluated by immunoblotting. Bar plots showing the relative expression level of COL12A1 protein in the indicated cell lines. Experiments were in triplicates. ****p < 0.0001, based on one-way analysis of variance (ANOVA). iCCA, intrahepatic cholangiocarcinoma; HCC, hepatocellular carcinoma; CHC, combined hepatocellular cholangiocarcinoma; FNH, focal nodular hyperplasia; TCGA, The Cancer Genome Atlas.

**Additional file 2: Fig. S2.** Forest plot to show univariate survival analysis results of iCCA patients (*n* = 60). iCCA, intrahepatic cholangiocarcinoma.

**Additional file 3: Fig. S3.** The overall methylation profile of COL12A1 gene in iCCA and adjacent nontumor tissue samples from TCGA (**A**) and GSE156299 (**B**) dataset, respectively. TCGA, The Cancer Genome Atlas; iCCA, intrahepatic cholangiocarcinoma.

**Additional file 4: Fig. S4.** Deciphering DNA methylation pattern of COL12A1 promoter region in iCCA and adjacent nontumor tissue samples. (**A**) Boxplots to show the methylation degree of CpG islands at COL12A1 enhancer region in iCCA and paired adjacent nontumor tissues samples from TCGA dataset. ns, *p* ≥ 0.05, using two-tailed paired Student's *t* test. (**B**) Boxplots to show the methylation degree of CpG islands at COL12A1 enhancer region in iCCA, intraductal tubulopapillary neoplasm of biliary duct (ITPN), intraductal papillary neoplasm of biliary duct (IPNB), and adjacent nontumor tissues samples from GSE156299 dataset. ns, *p* ≥ 0.05; *p < 0.05; **p < 0.01, using two-tailed unpaired Wilcox test. TCGA, The Cancer Genome Atlas; iCCA, intrahepatic cholangiocarcinoma.

**Additional file 5: Fig. S5.** Candidate miRNAs (miR-15a-5p, -497-5p, -15b-5p, -6838-5p, -16-5p, -195-5p and miR-424-5p) binding to wild-type COL12A1 3'UTR regions were predicted by TargetScanHuman database (version 8.0).

**Additional file 6: Fig. S6.** The expression patterns of the candidate miRNAs in clinical iCCA and adjacent nontumor liver tissue samples from TCGA dataset. (**A**) Boxplots showing that miRNAs (miR-424-5p, -424-3p, -497-5p, -15b-5p, -15a-5p, -16-5p, -195-5p and miR-16-5p) in iCCA and nontumor liver tissue samples from TCGA dataset. ns, *p* ≥ 0.05. **p < 0.01; ***p < 0.001, using two-tailed unpaired Student’s *t* test. (**B-C**) Heatmap shows the association of COL12A1 with miRNAs (miR-424-5p, -424-3p, -497-5p, -15b-5p, -15a-5p, -16-5p, -195-5p and miR-16-5p) in iCCA and nontumor samples from TCGA dataset. Numbers in heatmap denote Pearson coefficient (**B**) and statistical significance (**C**). Gallbladder or extrahepatic carcinoma tissue samples (*n* = 4) in TCGA cholangiocarcinoma dataset were excluded from our analysis. (**D**) MiR-424-5p in human iCCA (HuCCT1 and CCLP1 cell line) and normal human intrahepatic biliary cell (HIBEpiC) lines was determined by RT-PCR, respectively. RT-PCR, real-time PCR; TCGA, The Cancer Genome Atlas; iCCA, intrahepatic cholangiocarcinoma.

**Additional file 7: Table S1.** Correlation of COL12A1 expression with clinicopathological features of 60 iCCA patients.

**Additional file 8: Table S2.** The clinicopathological characteristics of 10 iCCA/HCC patients for COL12A1 expression profile analysis.

**Additional file 9: Table S3.** The designed primers of miR-424 promoter for targeted bisulfite sequencing.

## Data Availability

All data are included in the main text or online supplementary materials.

## Abbreviations

iCCA: Intrahepatic cholangiocarcinoma
COL12A1: Collagen type XII alpha 1 chain
HCC: Hepatocellular carcinoma
DNMT: DNA methyltransferase
TCGA: The Cancer Genome Atlas
GEO: Gene Expression Omnibus
RT-PCR: Real-time PCR
FNH: Focal nodular hyperplasia
CHC: Combined hepatocellular carcinoma
CpG: Cytosine–phosphate–guanine
ITPN: Intraductal tubulopapillary neoplasm of biliary duct
IPNB: Intraductal papillary neoplasm of biliary duct
