# Correction: Epigenetic dysregulation-mediated COL12A1 upregulation predicts worse outcome in intrahepatic cholangiocarcinoma patients

**DOI:** 10.1186/s13148-023-01455-3

**Published:** 2023-03-21

**Authors:** Zengwei Tang, Yuan Yang, Qi Zhang, Tingbo Liang

**Affiliations:** 1 Department of Hepatobiliary and Pancreatic Surgery, The First Affiliated Hospital, School of Medicine, Zhejiang University, Hangzhou, 310003 Zhejiang China; 2 Zhejiang Provincial Key Laboratory of Pancreatic Disease, The First Affiliated Hospital, School of Medicine, Zhejiang University, Hangzhou, 310003 Zhejiang China; 3 Zhejiang Clinical Research Center of Hepatobiliary and Pancreatic Diseases, Hangzhou, 310003 Zhejiang China; 4 Department of Hematology, Peking Union Medical College Hospital, Chinese Academy of Medical Sciences and Peking Union Medical College, Beijing, 100730 China; 5 Zhejiang University Cancer Center, Hangzhou, 310058 Zhejiang China

**Correction : Clinical Epigenetics (2023) 15:13**

**https://doi.org/10.1186/s13148-022-01413-5**

Following publication of the original article [1], the authors noticed an error in the Supplementary file. The online Additional File 4 does not belong to “the Supplementary file”. The typesetter has incorrectly uploaded Fig. 4 instead of Additional file 4: Fig. S4. This has been replaced with the correct file by publishing this correction. The ten pairs of HCC tissue samples used for immunoblotting (Fig. 2B) are the same as in our previous work [1]. The COL12A1 and NUP37 bands for the four pairs of HCC samples were collected from the same gel as well.

The original article has been corrected.

## Supplementary Information

**Additional file 4.**
