# Supplementary figures and images for: Epigenetic dysregulation-mediated COL12A1 upregulation predicts worse outcome in intrahepatic cholangiocarcinoma patients
Source: Clin Epigenetics. 2023 Jan 24;15:13. doi: 10.1186/s13148-022-01413-5 (PMC9875497; doi:10.1186/s13148-022-01413-5)

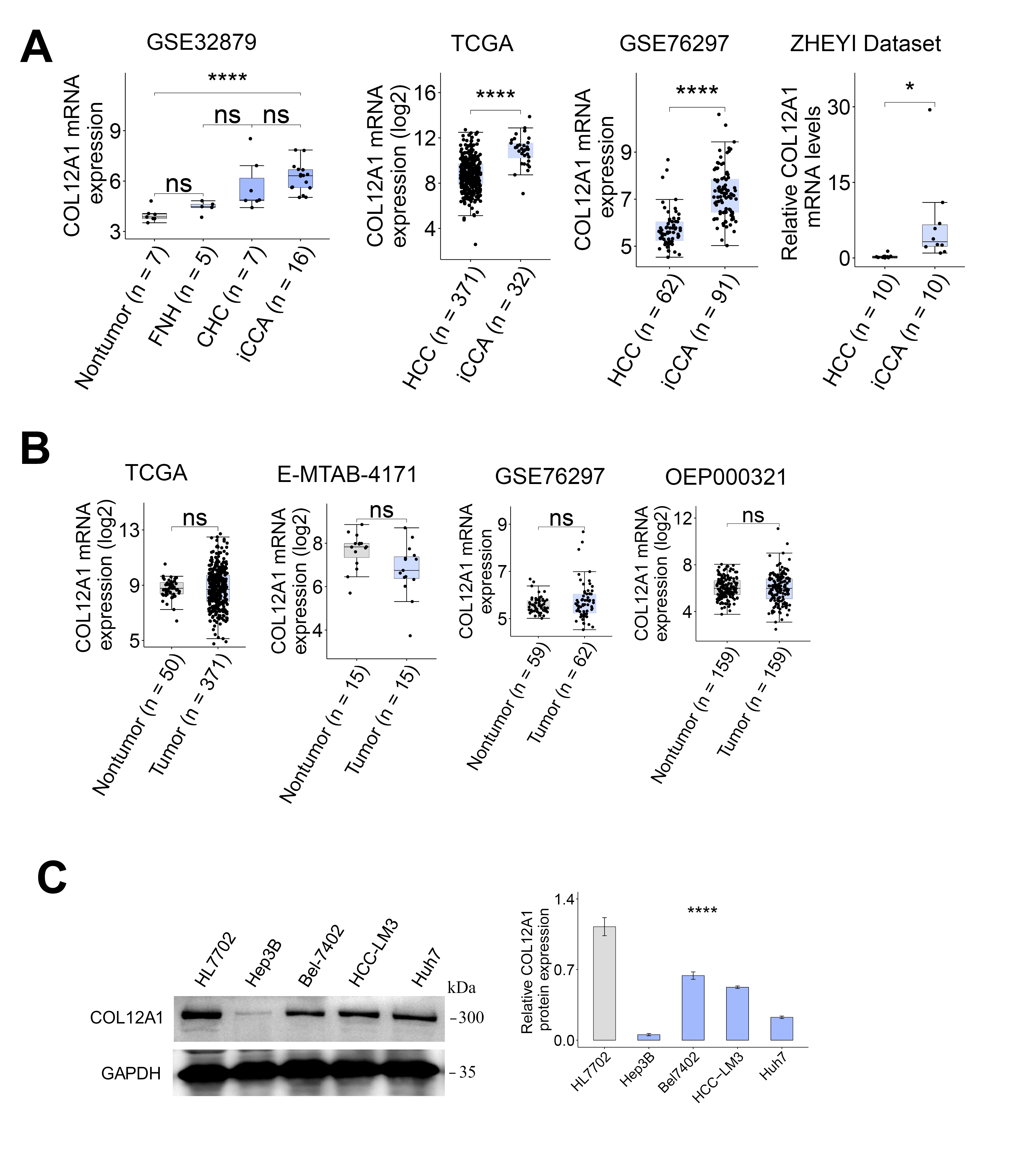

Supplement: Supplementary file 1 — Additional file 1: Fig. S1. COL12A1 mRNA expression is upregulated in clinical iCCA. (A) Boxplot to show COL12A1 mRNA expression levels in clinical iCCA, CHC, FNH and adjacent nontumor tissues samples from GSE32879 dataset. ns, p ≥ 0.05, **** p < 0.00001, using two-tailed unpaired Student's t test. Boxplot to show COL12A1 mRNA expression levels in clinical iCCA and HCC tissue samples form TCGA, GSE76297 or our in-house dataset, respectively. * p < 0.05; **** p < 0.00001, using two-tailed paired/unpaired Student's t test accordingly. Gallbladder or extrahepatic carcinoma tissue samples (n = 4) in TCGA cholangiocarcinoma dataset were excluded from our analysis. (B) Boxplot to show COL12A1 mRNA expression levels in clinical HCC and nontumor liver tissue samples from TCGA, E-MTAB-4171, GSE76297 or OEP000321 dataset, respectively. ns, p ≥ 0.05, using two-tailed paired/unpaired Student's t test accordingly. (C) COL12A1 protein levels in whole cell lysate of human HCC cell lines (including Hep3B, Bel-7402, HCC-LM3, and Huh7) and normal liver cell line HL7702 were evaluated by immunoblotting. Bar plots showing the relative expression level of COL12A1 protein in the indicated cell lines. Experiments were in triplicates. ****p < 0.0001, based on one-way analysis of variance (ANOVA). iCCA, intrahepatic cholangiocarcinoma; HCC, hepatocellular carcinoma; CHC, combined hepatocellular cholangiocarcinoma; FNH, focal nodular hyperplasia; TCGA, The Cancer Genome Atlas. [file 13148_2022_1413_MOESM1_ESM.jpg]

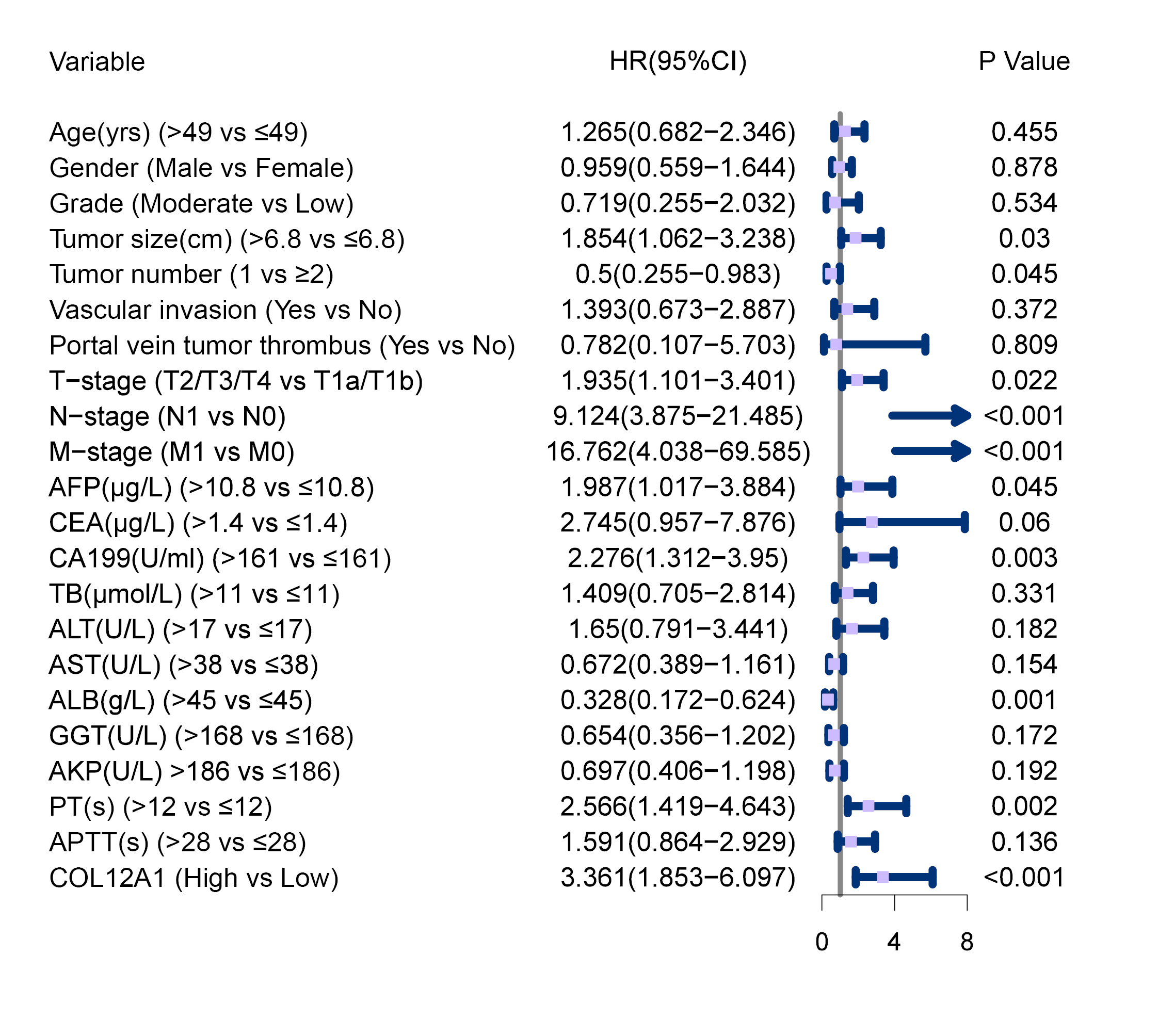

Supplement: Supplementary file 2 — Additional file 2: Fig. S2. Forest plot to show univariate survival analysis results of iCCA patients (n = 60). iCCA, intrahepatic cholangiocarcinoma. [file 13148_2022_1413_MOESM2_ESM.jpg]

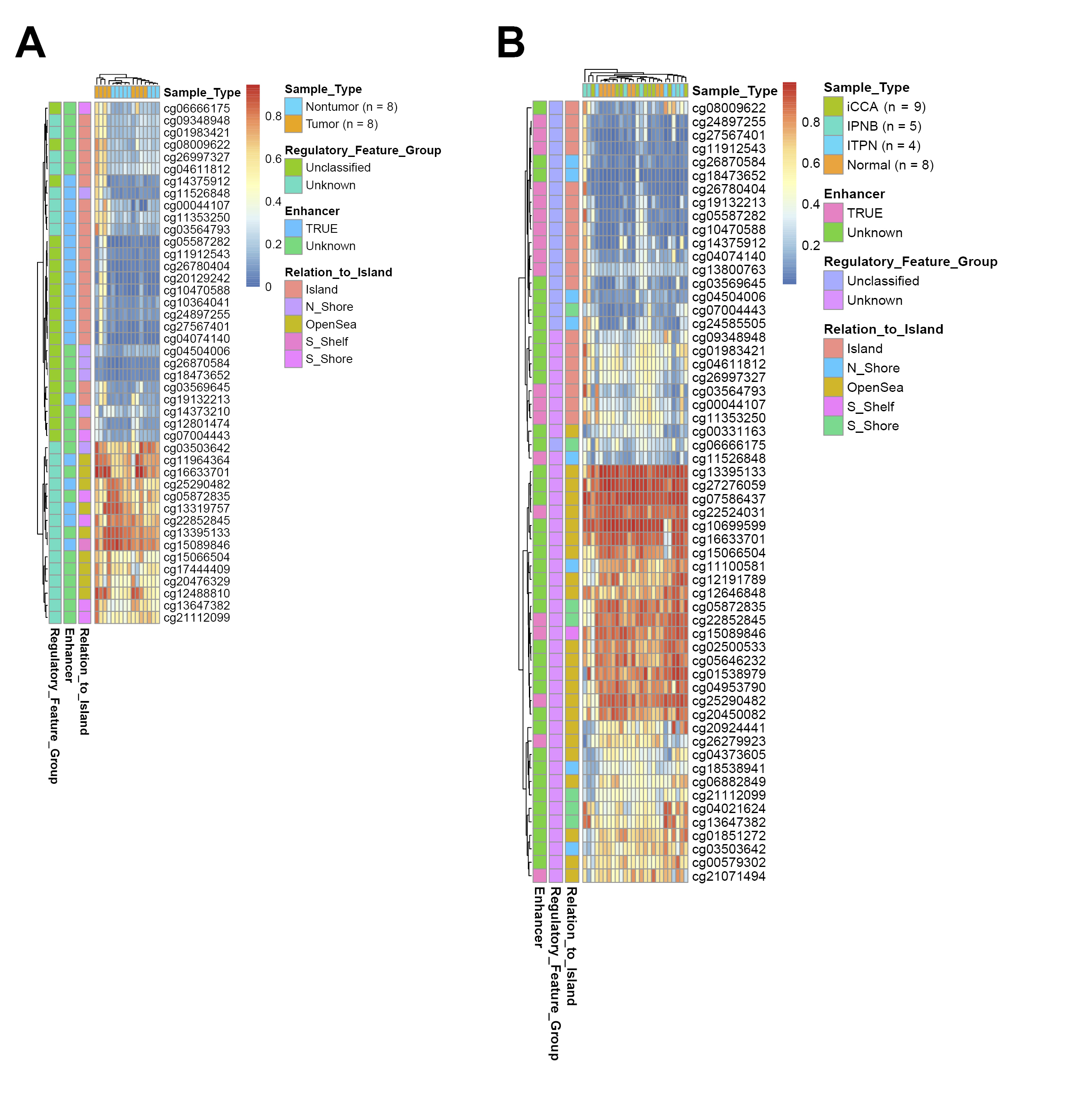

Supplement: Supplementary file 3 — Additional file 3: Fig. S3. The overall methylation profile of COL12A1 gene in iCCA and adjacent nontumor tissue samples from TCGA (A) and GSE156299 (B) dataset, respectively. TCGA, The Cancer Genome Atlas; iCCA, intrahepatic cholangiocarcinoma. [file 13148_2022_1413_MOESM3_ESM.jpg]

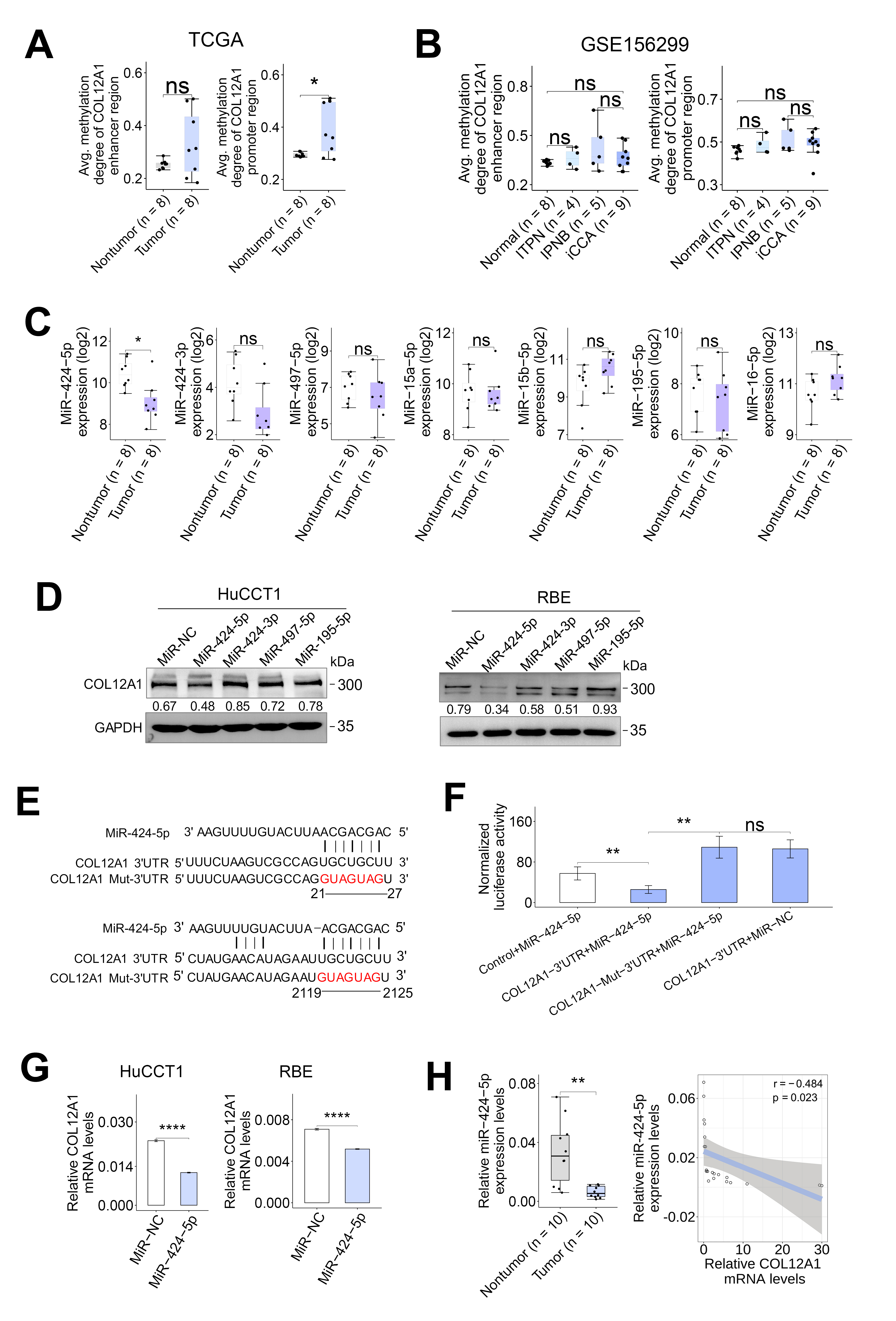

Supplement: Supplementary file 4 — Additional file 4: Fig. S4. Deciphering DNA methylation pattern of COL12A1 promoter region in iCCA and adjacent nontumor tissue samples. (A) Boxplots to show the methylation degree of CpG islands at COL12A1 enhancer region in iCCA and paired adjacent nontumor tissues samples from TCGA dataset. ns, p ≥ 0.05, using two-tailed paired Student's t test. (B) Boxplots to show the methylation degree of CpG islands at COL12A1 enhancer region in iCCA, intraductal tubulopapillary neoplasm of biliary duct (ITPN), intraductal papillary neoplasm of biliary duct (IPNB), and adjacent nontumor tissues samples from GSE156299 dataset. ns, p ≥ 0.05; *p < 0.05; **p < 0.01, using two-tailed unpaired Wilcox test. TCGA, The Cancer Genome Atlas; iCCA, intrahepatic cholangiocarcinoma. [file 13148_2022_1413_MOESM4_ESM.jpg]

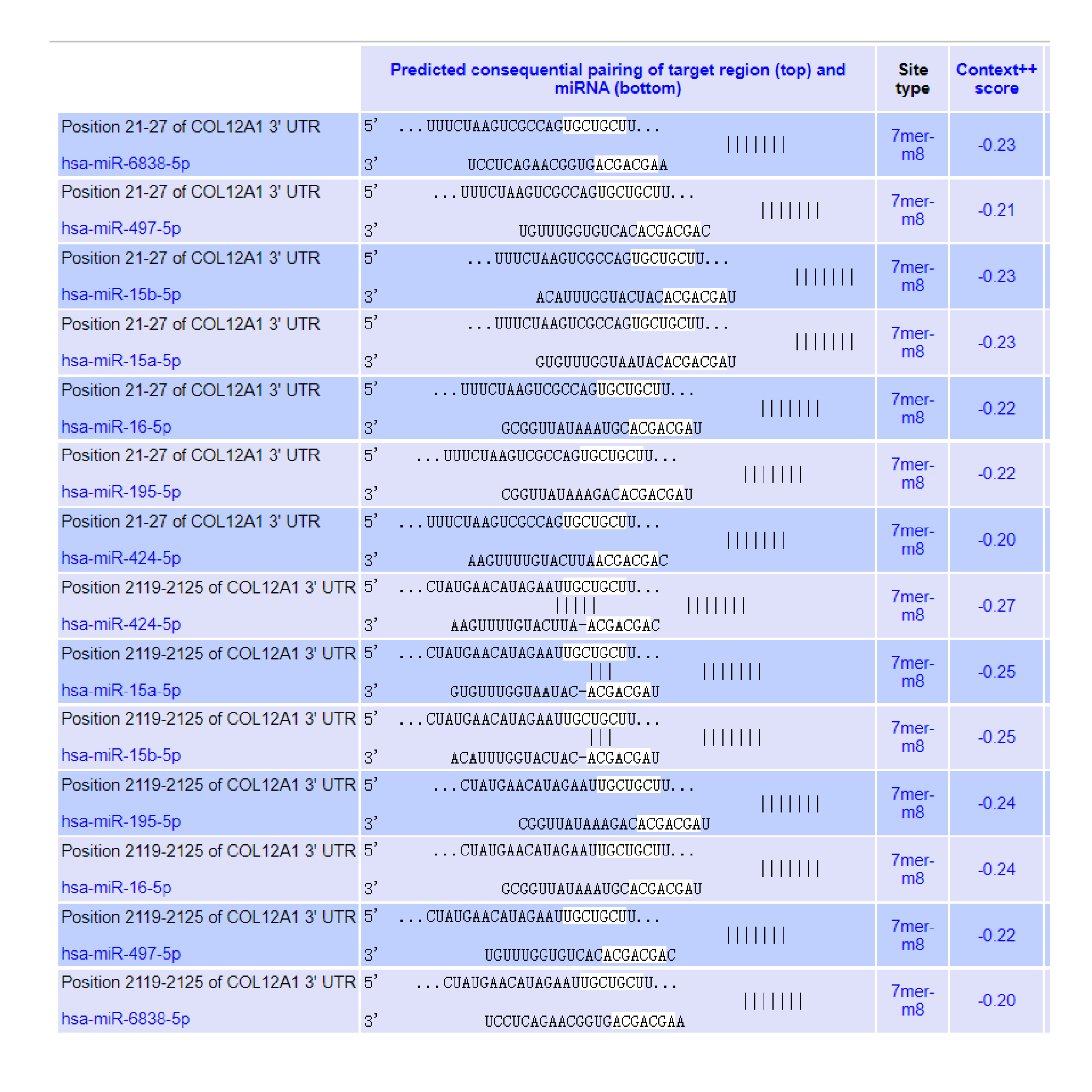

Supplement: Supplementary file 5 — Additional file 5: Fig. S5. Candidate miRNAs (miR-15a-5p, -497-5p, -15b-5p, -6838-5p, -16-5p, -195-5p and miR-424-5p) binding to wild-type COL12A1 3'UTR regions were predicted by TargetScanHuman database (version 8.0). [file 13148_2022_1413_MOESM5_ESM.jpg]

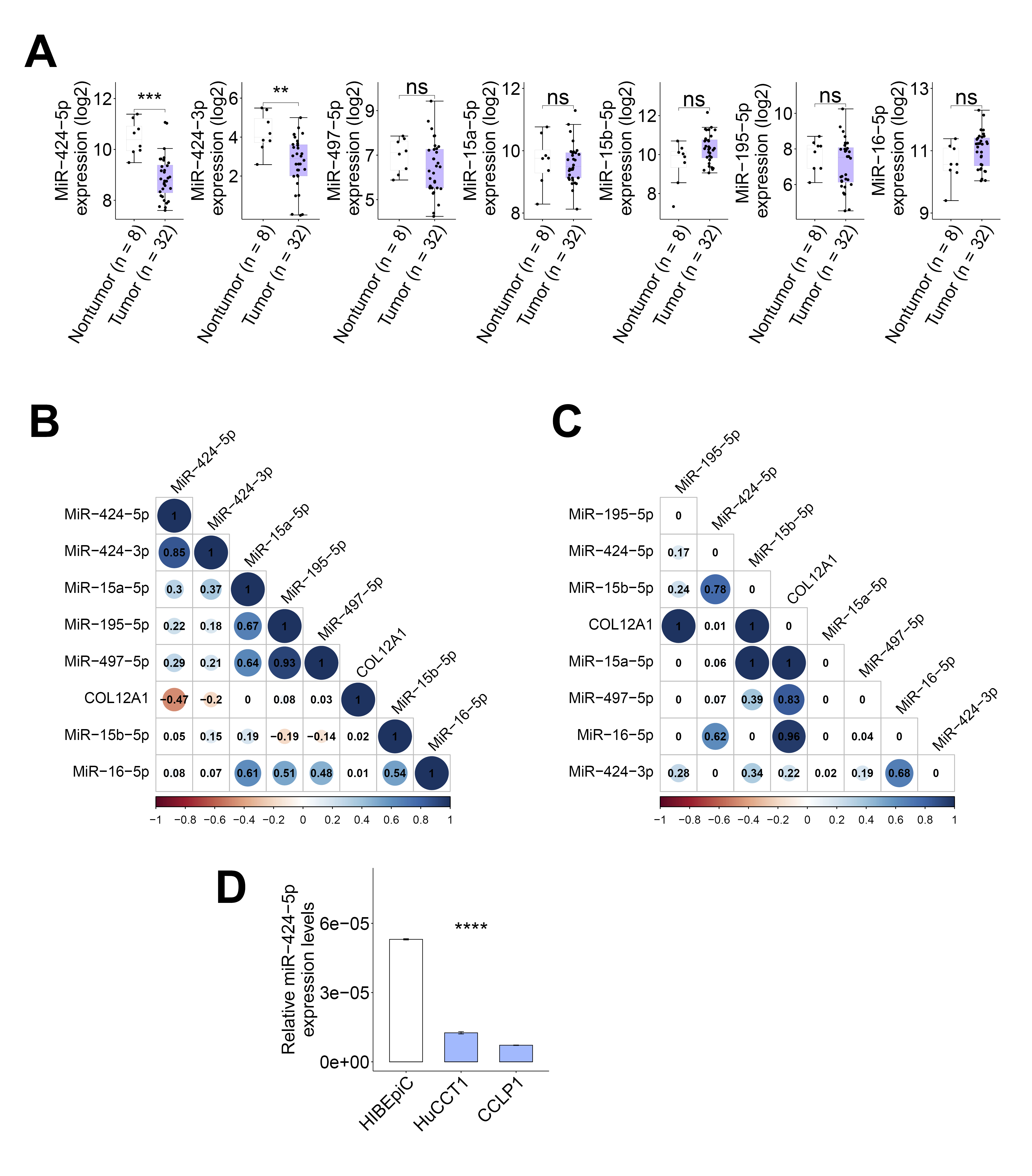

Supplement: Supplementary file 6 — Additional file 6: Fig. S6. The expression patterns of the candidate miRNAs in clinical iCCA and adjacent nontumor liver tissue samples from TCGA dataset. (A) Boxplots showing that miRNAs (miR-424-5p, -424-3p, -497-5p, -15b-5p, -15a-5p, -16-5p, -195-5p and miR-16-5p) in iCCA and nontumor liver tissue samples from TCGA dataset. ns, p ≥ 0.05. **p < 0.01; ***p < 0.001, using two-tailed unpaired Student’s t test. (B-C) Heatmap shows the association of COL12A1 with miRNAs (miR-424-5p, -424-3p, -497-5p, -15b-5p, -15a-5p, -16-5p, -195-5p and miR-16-5p) in iCCA and nontumor samples from TCGA dataset. Numbers in heatmap denote Pearson coefficient (B) and statistical significance (C). Gallbladder or extrahepatic carcinoma tissue samples (n = 4) in TCGA cholangiocarcinoma dataset were excluded from our analysis. (D) MiR-424-5p in human iCCA (HuCCT1 and CCLP1 cell line) and normal human intrahepatic biliary cell (HIBEpiC) lines was determined by RT-PCR, respectively. RT-PCR, real-time PCR; TCGA, The Cancer Genome Atlas; iCCA, intrahepatic cholangiocarcinoma. [file 13148_2022_1413_MOESM6_ESM.jpg]
