# Supplementary material for: Epigenetic dysregulation-mediated COL12A1 upregulation predicts worse outcome in intrahepatic cholangiocarcinoma patients
Source: Clin Epigenetics. 2023 Jan 24;15:13. doi: 10.1186/s13148-022-01413-5 (PMC9875497; doi:10.1186/s13148-022-01413-5)
Supplement: Supplementary file 7 — Additional file 7: Table S1. Correlation of COL12A1 expression with clinicopathological features of 60 iCCA patients. [file 13148_2022_1413_MOESM7_ESM.pdf]

Table S1. Correlation of COL12A1 expression with clinicopathological features of 60 iCCA patients

Variable		COL12A1		*P* value
		Low	High	
Age (yrs)	≤ 49	4	10	0.756
	＞49	16	30	
Gender	Female	7	16	0.707
	Male	13	24	
Histological grade	Low	2	3	1.000
	Moderate/ Moderate-Low	18	37	
Tumor size (cm)	≤ 6.8	16	22	0.058
	＞6.8	4	18	
Tumor number	1	3	10	0.513
	≥ 2	17	30	
Vascular invasion	No	18	33	0.704
	Yes	2	7	
Portal vein tumor thrombus	No	19	39	1.000
	Yes	1	1	
AFP (μg/L)	≤ 10.8	17	30	0.513
	＞10.8	3	10	
CEA (μg/L)	≤ 1.4	1	4	0.456
	＞1.4	19	36	
CA199 (U/mL)	≤ 161	13	22	0.201
	＞161	7	18	
TB (μmol/L)	≤ 11	3	8	0.736
	＞11	17	32	
ALT (U/L)	≤ 17	3	7	1.000
	＞17	17	33	
AST (U/L)	≤ 11	11	27	0.344
	＞11	9	13	
ALB (g/L)	≤ 45	11	31	0.073
	＞45	9	9	
GGT (U/L)	≤ 168	13	30	0.418
	＞168	7	10	
AKP (U/L)	≤ 186	11	26	0.453
	＞186	9	14	
PT (s)	≤ 12	15	23	0.185
	＞12	5	17	
APTT (s)	≤ 28	16	27	0.311
	＞28	4	13	

Table S1 continued

T-stage (AJCC8th)	T1a/T1b	16	21	0.039
	T2/T3/T4	4	19	
N-stage (AJCC8th)	N0	19	28	0.043
	N1	1	12	
M-stage (AJCC8th)	M0	20	37	0.544
	M1	0	3	
TNM staging	IA/IB	15	16	0.022
	II/III/IV	5	24	
