# Supplementary material for: Epigenetic dysregulation-mediated COL12A1 upregulation predicts worse outcome in intrahepatic cholangiocarcinoma patients
Source: Clin Epigenetics. 2023 Jan 24;15:13. doi: 10.1186/s13148-022-01413-5 (PMC9875497; doi:10.1186/s13148-022-01413-5)
Supplement: Supplementary file 8 — Additional file 8: Table S2. The clinicopathological characteristics of 10 iCCA/HCC patients for COL12A1 expression profile analysis. [file 13148_2022_1413_MOESM8_ESM.pdf]

Table S2. The clinicopathological characteristics of 10 iCCA/HCC patients for COL12A1 expression profile analysis

Variable/clinical outcome	No. iCCA pts (n = 10)	No. HCC pts (n = 10)
Gender (F:M)	6:4	3:7
Age, mean (yrs, range)	64.6 (45-84)	57.5 (34-72)
Histological grade		
Low	5	0
Moderate/Moderate-Low	5	9
Well	0	1
T-stage (AJCC^{8th})		
T1a/T1b	5/4	0/2
T2	1	8
T3	0	0
T4	0	0
N-stage (AJCC^{8th})		
N0	6	4
N1	4	0
Nx	0	6
M-stage (AJCC^{8th})		
M0	10	10
M1	0	0

iCCA, intrahepatic cholangiocarcinoma; HCC, hepatocellular carcinoma
