# Supplementary material for: Epigenetic dysregulation-mediated COL12A1 upregulation predicts worse outcome in intrahepatic cholangiocarcinoma patients
Source: Clin Epigenetics. 2023 Jan 24;15:13. doi: 10.1186/s13148-022-01413-5 (PMC9875497; doi:10.1186/s13148-022-01413-5)
Supplement: Supplementary file 9 — Additional file 9: Table S3. The designed primers of miR-424 promoter for targeted bisulfite sequencing. [file 13148_2022_1413_MOESM9_ESM.pdf]

| **Table S3. The designed primers of miR-424 promoter for targeted bisulfite sequencing** | | | |
|---|---|---|---|
| Region | Primer sequences | Chromosome location of targeted region (bp) | No. CpG sites |
| Locus 1 | Forward: AAAATYGTATGTTATTTTTGGTTTTATTYGAAAATGG Reverse: AACCCTTCCTTCCTCCCTATCTCCCT | chrX:33683989-133684023 | 48 |
| Locus 2 | Forward: GAGAGGTTTTYGGAGTYGTTTTAGAA Reverse: TTCCCTACAACTCCTCTCCTCCCCTC | chrX:133683228-133683268 | |
| Locus 3 | Forward: AGGGTYGAGAAGGGTATTGGGYGTTTAGAG Reverse: CAAAAAACTTCRCTCAACCACCTTAC | chrX:133683677-133683702 | |
| Locus 4 | Forward: TGAGATGGTTTGGGAAAGGAAATAGG Reverse: CCTCTCTACCTAAAAACTTAATAAAATAAC | chrX:133684115-133684140 | |
