# Supplementary figures and images for: Correction: Epigenetic dysregulation-mediated COL12A1 upregulation predicts worse outcome in intrahepatic cholangiocarcinoma patients
Source: Clin Epigenetics. 2023 Mar 21;15:46. doi: 10.1186/s13148-023-01455-3 (PMC10031879; doi:10.1186/s13148-023-01455-3)

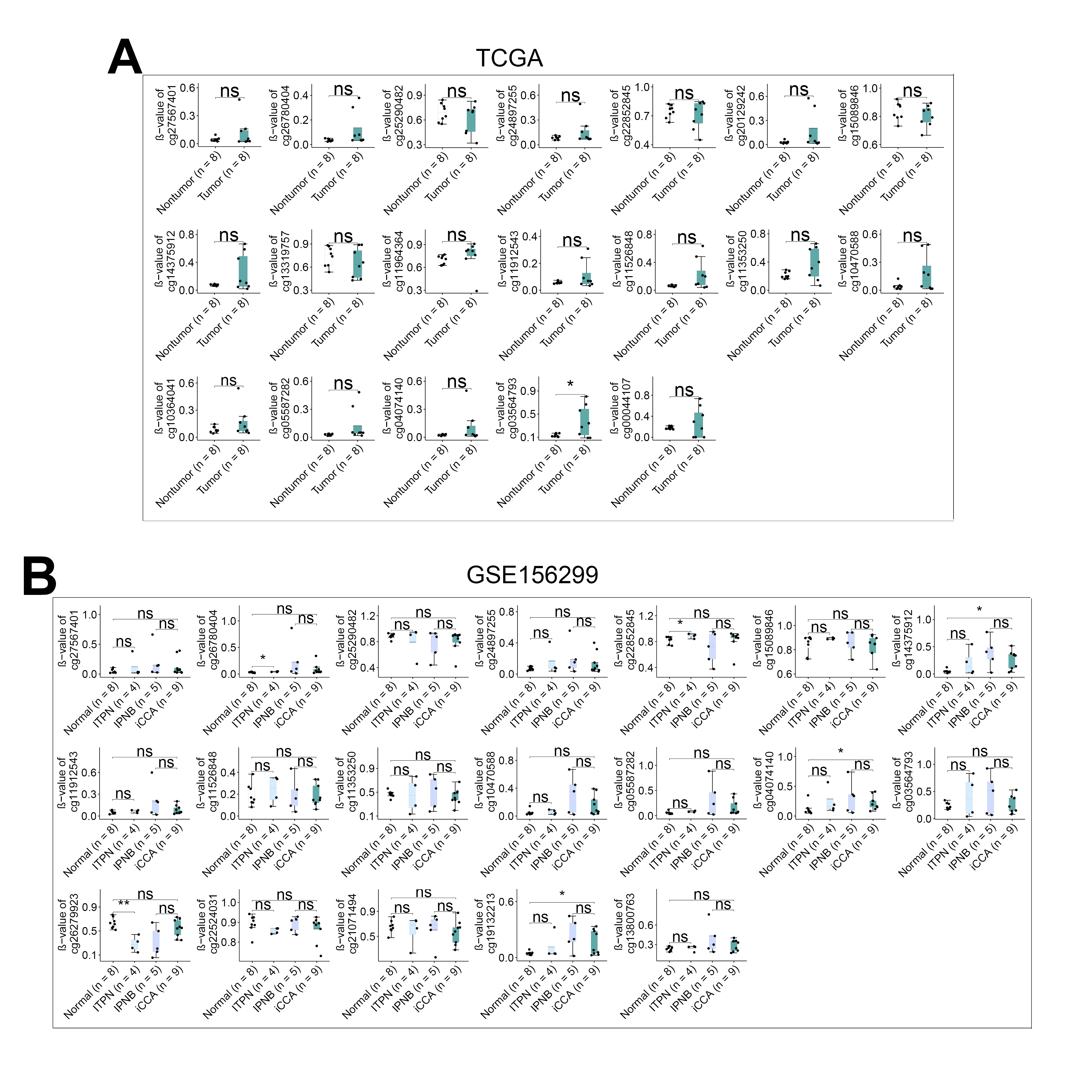

Supplement: Supplementary file 1 — Additional file 4. [file 13148_2023_1455_MOESM1_ESM.jpg]
